# Differential Regulation of the STING Pathway in Human Papillomavirus–Positive and -Negative Head and Neck Cancers

**DOI:** 10.1158/2767-9764.CRC-23-0299

**Published:** 2024-01-16

**Authors:** Emma L. Saulters, Paul T. Kennedy, Rachel J. Carter, Abdullah Alsufyani, Terence M. Jones, John F. Woolley, Lekh N. Dahal

**Affiliations:** 1 Department of Pharmacology and Therapeutics, University of Liverpool, Liverpool, United Kingdom.; 2 Department of Molecular and Clinical Cancer Medicine, University of Liverpool, Liverpool, United Kingdom.

## Abstract

Squamous cell carcinomas, which arise from the cells that line the mucosal surfaces of the head and neck, represent the most common type of head and neck cancers (HNSCC). Human papillomavirus (HPV) infection has been strongly associated with the development of oropharyngeal cancers, which are cancers that occur in the back of the throat, including the tonsils and base of the tongue. HNSCCs with and without HPV infection have distinct pathology, with HPV-positive patients having higher levels of immune infiltration, activation in the tumor microenvironment and better response to radiation and chemotherapy. It is, however, unclear whether HPV infection in HNSCCs has the potential to activate innate-immune sensing pathways and if these cancers possess intrinsic immunogenicity associated with HPV infection. Here we investigate the innate immune stimulator of interferon genes (STING) pathway and immune responses to STING activation in HNSCCs and uncover fundamental differences in the regulation of this pathway in cell lines versus primary human clinical specimens. We show that while STING is differentially expressed in HPV-positive and -negative HNSCC cell lines, they exhibit a gross functional defect in signaling through this pathway. However, STING activation in immune cell populations generated immune signatures predicted to elicit useful tumoricidal mechanisms. In contrast, IHC analysis of human tissue microarrays revealed enhanced STING expression in HPV-related tumors and high intratumoral expression of STING correlated with increased survival.

**Significance:**

STING is an important innate immune sensor of cytosolic DNA, inducing essential antiviral and antitumoral responses. This research shows that STING expression is enhanced in HPV-positive HNSCC patient tissue, with high intratumoral STING expression correlating with increased survival. In addition, STING activation in immune cell populations augmented antitumoral effects against HNSCCs, suggesting patients may benefit from the use of STING agonists in combination with traditional therapies.

## Introduction

Head and neck cancers, of which the majority are squamous cell carcinomas (HNSCC) represent a heterogeneous cluster of aggressive malignancies that constitute the seventh most common cancer worldwide (1, 2). HNSCCs can be classified into two etiologically distinct phenotypes, dependent upon oncogenic human papillomavirus (HPV) infection (3). Conventional primary risk factors for HNSCCs are tobacco- and alcohol-induced mutations, and in recent decades, HPV has been identified as a new risk factor for these cancers (4). Evidence of HPV involvement in HNSCCs was first reported in 1983 (5, 6) and infection with high-risk subtypes (predominantly HPV16) was officially classified as a causative factor of oropharyngeal SCCs (OPSCC) in 2000 (7). HPV is a circular double-stranded DNA virus that can integrate into host DNA of cells within the crypts of the tonsil and base of tongue (8). The virus can initiate tumorigenesis by inactivating the tumor suppressor proteins p53 and retinoblastoma protein (pRb) through the action of the E6 and E7 oncogenes, respectively (9). The incidence of HPV^+^ OPSCCs has significantly increased over the past 30 years and now accounts for 50%–70% of all OPSCCs in Western countries (10), representing an increasing global health concern.

For reasons not currently well understood, HNSCC patients with HPV^+^ tumors have a better outcome in terms of both survival and reduced risk of recurrence (11, 12). HPV^+^ patients generally show enhanced responses to traditional treatment regimens (13), but the precise underlying mechanisms involved in producing favorable outcomes remain unknown. HPV^+^ and HPV^−^ HNSCCs have pathologically distinct tumors; with HPV^+^ patients known to have higher levels of immune infiltration and activation in the tumor microenvironment (TME; ref. 14). Several components of the innate immune-sensing pathway can be activated following recognition of viral nucleotide compounds emitting damage-associated molecular patterns, which can lead to the development of antitumor immunity (15). It is not known whether HPV infection has the potential to modulate the interface between innate immune sensors and adaptive immune cell responses, which are crucial for the therapeutic benefit of patients.

One important innate immune-sensing pathway is the stimulator of interferon genes (STING); a central component of our response to infection, sensing parasitic, bacterial, or viral DNA in the cytosol and inducing type-I interferons (IFNα and IFNβ) in response (16, 17). STING is activated directly by cytosolic DNA or 2′3′-cGAMP produced by cyclic GMP-AMP synthase (cGAS) following detection of cytosolic double-stranded DNA (16, 18). Activated STING undergoes a conformational change that allows it to translocate from the endoplasmic reticulum (ER) to the Golgi via ER–Golgi intermediate compartments, where tank binding kinase 1 (TBK1) and IκB kinase (IKK) are recruited and activated. At perinuclear regions, interferon regulatory factor 3 (IRF3) and NF-κB are recruited and activated by the STING complex, allowing these transcription factors to enter into the nucleus and induce production of type-I IFNs (19, 20).

While STING was originally considered to be a key regulator of antiviral immune responses (21), the immune boosting adjuvant properties elicited by synthetic STING agonists are being extensively investigated in preclinical and clinical studies with a view to identify novel STING agonists of therapeutic relevance for the treatment of cancer (refs. 22–24; NCT03956680, NCT04144140). The role STING plays in HNSCCs remains to be elucidated, but its multifunctional role in DNA sensing, including HPV detection (25), suggests that the STING pathway may have important effects in the two HNSCC phenotypes. Here we document an extensive characterization of the STING pathway in human HNSCC cell lines and primary human tissues and reveal fundamental differences in the STING protein expression, downstream signaling, and functional modulation of immune responses by the HPV^+^ and HPV^−^ tumor types.

## Materials and Methods

### Cell Lines, Culture Conditions, and Drugs

Experiments were conducted in seven human HNSCC cell lines of both negative and positive HPV status obtained from ATCC and authenticated by short tandem repeat (STR) profiling. They were routinely checked for *Mycoplasma* contamination by qPCR. UM-SCC-1 (RRID:CVCL_7707), UM-SCC-11B (RRID:CVCL_7716), UM-SCC-81B (RRID:CVCL_7784) (HPV^−^), UM-SCC-47 (RRID:CVCL_7759) and UPCI:SCC-090 (RRID:CVCL_1899) (HPV^+^) were cultured in DMEM supplemented with GlutaMAX (Gibco), 10% FBS (Invitrogen), and 1x nonessential amino acids (NEAA; Gibco). FaDu (RRID:CVCL_1218; HPV^−^) and UPCI:SCC-154 (RRID:CVCL_2230; HPV^+^) cells were cultured in modified Eagle's medium (MEM; Gibco) supplemented with GlutaMAX (Gibco), 10% FBS, 1x NEAA, and 3% sodium bicarbonate (Gibco). HeLa (RRID:CVCL_0030) and human monocytic THP-1 (RRID:CVCL_0006) were used as control cell lines and were cultured in DMEM and RPMI1640 media (Gibco) supplemented with 10% FBS and 100 U/mL of penicillin and 100 µg/mL streptomycin (Gibco), respectively. All cells were cultured at 37°C in a 5% CO_2_ humidified incubator and passaged three to five times between thawing and use in the described experiments. The STING agonist ML RR-S2 CDA (CDA; MedChemExpress, catalog no. HY-12885) was used at indicated concentrations.

### STING Overexpression and Knockdown

A total of 1.5 × 10^5^ HNSCC cells were plated in 6-well plates and left to adhere overnight. Cells were transfected with pcDNA3.1 STING plasmid (GenScript, cloneID OHu16678D) using Viafect (Promega, catalog no. E498A) incubated in Opti-MEM (Gibco). RNA and proteins were collected after 2- and 3-day incubations, respectively. Confluent HNSCC cells were transfected with 15 nmol/L STING or control targeting siRNAs (Qiagen) by the use of Dharmafect I (Horizon Discovery, catalog no. T-2001–01) following manufacturer's protocol. Cells were collected 72 hours posttransfection for further experiments.

### RNA Isolation and RT-qPCR

Total RNA was isolated using the total RNA Miniprep kit (NEB, catalog no. T2010S) following the manufacturer's protocol. RNA (5 µg) was reverse transcribed into cDNA using M-MLV reverse transcriptase (Invitrogen, catalog no. 28025013) and random primers (NEB, catalog no. S1330S). cDNA concentrations were normalized and RT-qPCR was performed using specific primers and GoTaq qPCR master mix (Promega, catalog no. A6001) according to the manufacturer's specifications on the AriaMX Real-Time qPCR machine (Agilent). Expression of target mRNAs was normalized to β-actin and relative gene expression was calculated using Δ*C*_t_ and 2^−ΔΔ^*^C^*_t_.

#### Primers

Published primer pairs were used to assess all genes of the HPV genome (26).

**Table:** 

HPV E1	fwrd-5′-AGTAGAGCTGCAAAAAGGAGATTA-3′ rev-5′-CTGACTACATGGTGTTTCAGTCTC-3′
HPV E2	fwrd-5′- AACGAAGTATCCTCTCCTGAAATTATTAG-3′ rev-5′-CCAAGGCGACGGCTTTG-3′
HPV E4	fwrd-5′-GACTATCCAGCGACCAAGATCAG-3′ rev-5′-CTGAGTCTCTGTGCAACAACTTAGTG-3′
HPV E5	fwrd-5′-GCGACGTGAGAGCAACG-3′ rev-5′-AGGGGTTTCCGGTGTCTGG-3′
HPV E6	fwrd-5′-GAGAACTGCAATGTTTCAGGACC-3′ rev-5′-TGTATAGTTGTTTGCAGCTCTGTGC-3′
HPV E7	fwrd-5′-AAGTGTGACTCTACGCTTCGGTT-3′ rev-5′-GCCCATTAACAGGTCTTCCAAA-3′
HPV L1	fwrd-5′-TTAGGTGTGGGCATTAGTGG-3′ rev-5′-TCCCCTATAGGTGGTTTGCA-3′
HPV L2	fwrd-5′-GACCCTGCTTTTGTAACCACTC-3′ rev-5′-ATGCTGGCCTATGTAAAGCAAC-3′
STING	fwrd-5′-GAGCAGGCCAAACTCTTCTG-3′ rev-5′-TGCCCACAGTAACCTCTTCC-3′
IFNβ	fwrd-5′-TAGCACTGGCTGGAATGAG-3′ rev-5′-GTTTCGGAGGTAACCTGTAAG-3′
β-actin	fwrd-5′-TCACCCACACTGTGCCCATCTACGA-3′ rev-5′-CAGCGGAACCGCTCATTGCCAATGG-3′

### Western Blot Analysis

Cells were lysed with RIPA buffer (Sigma-Aldrich, catalog no. R0278) containing protease inhibitors (Sigma-Aldrich, catalog no. P8340) and centrifuged at 13,000 rpm at 4°C for 30 minutes. Protein concentration was quantified using the Pierce BCA Kit (Thermo Fisher Scientific, catalog no. 23227). Equal amounts of protein were separated on 10% SDS-PAGE gels and electroporated onto nitrocellulose membranes. Membranes were blocked with 5% BSA or 5% milk (Sigma-Aldrich) diluted in Tris-buffered saline-Tween-20 (TBS-T) for 1 hour at room temperature. Proteins were incubated at 4°C overnight with primary antibodies against phospho-STING (1:2,000, Cell Signaling Technology), STING (1:3,000, Cell Signaling Technology), phospho-IRF3 (1:2,000, Cell Signaling Technology), IRF3 (1:3,000, Cell Signaling Technology), phospho-TBK1 (1:2,000, Cell Signaling Technology), TBK1 (1:3,000, Cell Signaling Technology), MAVS (1:3,000, Cell Signaling Technology), MyD88 (1:3,000, Cell Signaling Technology), phospho-STAT1 (1:2,000, ProteinTech), STAT1 (1:3,000, ProteinTech), and β-actin (1:25,000 Sigma-Aldrich; Information on antibodies used in Supplementary Table S1). Membranes were incubated with secondary antibodies (1:3,000) for 1 hour at room temperature before visualization with Immobilon chemiluminescent HPR substrate (Millipore, catalog no. WBKLS0500) on the ChemiDoc Imaging System (Bio-Rad). Densitometric analysis of protein expression was performed using ImageJ (RRID:SCR_003070). Background signal was subtracted from each band intensity before target protein expression normalization to the loading control.

### Multiplex ELISA

Culture supernatants were collected for cell lines ± 10 µg/mL CDA stimulation for 6 hours and stored at −80°C. Cell culture supernatants were transferred into the inlet ports of CodePlex innate immune chips (Isoplexis, catalog no. S-Panel-2L03) following the manufacturer's protocol. The chips were loaded and run on the Isolight machine and data were processed using the IsoSpeak software (Isoplexis). Analysis was performed in R and visualized using *factoextra* (RRID:SCR_016692; ref. 27) and *corrplot* (RRID:SCR_024683; ref. 28) packages.

### Next-Generation Sequencing and Differential Gene Expression Analysis

Total RNA was isolated from HNSCC cells using the total RNA Miniprep kit (NEB) according to the manufacturer's protocol. NanoDrop spectrophotometer (Thermo Fisher Scientific) and agarose bleach gels were used to confirm RNA quality and the samples were sent for library preparation (QuantSeq Library Prep Kit) and sequencing (NextSeq200 using single -nd 1 × 100-bp read length and 16 million reads per sample; Glasgow Proteomics, University of Glasgow). Quant-seq was performed using three biological replicates.

Data were processed, normalized, and underwent quality control checks. Differential gene expression analysis between HPV^+^ and HPV^−^ HNSCC cell line normalized counts was performed using the R package *DESeq2* (RRID:SCR_015687; ref. 29), using the Wald test to calculate *P* values. Genes with an adjusted *P* value < 0.01 following a Benjamini–Hochberg test and a log_2_ fold change (log_2_FC) of 1 were considered differentially expressed genes. Gene ontology (GO) analysis (RRID:SCR_002811) and gene-set enrichment analysis (GSEA; RRID:SCR_003199) of differentially expressed Hallmark pathways were implemented and visualized using the R package *clusterProfiler* (RRID:SCR_016884; ref. 30).

### PBMC Isolation

Human PBMCs were purified by lymphoprep (StemCell Technologies, catalog no. 07851) density gradient centrifugation from healthy donor leukocyte cones (purchased from NHS Blood and Transplant, Speke, Liverpool, United Kingdom).

### STING-mediated Cytokine ELISAs

HNSCC cells (2 × 10^5^) were seeded in 24-well plates overnight. The next day, PBMCs (3 × 10^6^) were cocultured with the HNSCC cells with/without 10 µg/mL CDA for 2 and 6 hours. For transwell cocultures, 2 × 10^5^ HNSCC cells were seeded into the bottom compartment of a 24-well plate overnight. PBMCs (3 × 10^6^) were seeded into the top 0.4-µm ThinCert cell culture insert (Greiner Bio-One, catalog no. 665641) the next day and cultured for 6 hours ± 10 µg/mL CDA. Supernatants were harvested and the levels of IFNβ, IL6, TNFα, and IL1β were assessed with human type-I IFN (Biorbyt, catalog no. orb561974), TNFα (Invitrogen, catalog no. 88–7346), IL6 (Invitrogen, catalog no. 88–7066), and IL1β (Invitrogen, catalog no. 88–7261) ELISA kits following the manufacturer's instructions.

### Mass Cytometry

Antibodies were either purchased preconjugated to metal isotopes (Fluidigm) or conjugated in house using Maxpar Antibody Labeling Kits (Standard Biotools) according to the manufacturer's protocol. HNSCC cells (3 × 10^5^) were seeded in 12-well plates overnight. The next day, PBMCs (5 × 10^6^) were cocultured with the HNSCC cells ± 10 µg/mL CDA for 6 hours before PBMC collection for mass cytometry processing and staining. PBMCs were incubated with Cell-ID Cisplatin (Fluidigm, catalog no. 201195) suspended in PBS for 5 minutes to discriminate viable/dead cells. Cells were washed in PBS and resuspended in cell staining buffer (CSB, Fluidigm, catalog no. 201068). PBMC replicates were stained with combinations of ^89^Y-, ^106^Cd-, ^110^Cd-, and ^115^Cd-CD45 for 30 minutes on ice. Cells were washed twice in CSB and pooled together according to barcoding strategy and then stained with a cocktail of cell surface antibodies (Supplementary Table S2) diluted in CSB for 30 minutes at room temperature. Cells were washed twice in CSB and resuspended in 1x Fix I buffer (Fluidigm, catalog no. 201065) for 10 minutes at room temperature. Cells were centrifuged and resuspended in 300 µL of Fix and Perm Buffer (Fluidigm, catalog no. 201067) per 3 × 10^6^ cells overnight. Cells were permeabilized with FoxP3 Fix/Perm buffer (Fluidigm, catalog no. 00–5123–43) for 40 minutes at room temperature, washed twice with 1x Perm buffer at 800 × *g* for 5 minutes, and incubated with 100 µL of the intracellular antibody cocktail for 30 minutes at room temperature. Samples were washed twice in 1x Perm buffer and incubated in a solution of Intercolator-Ir (Fluidigm, catalog no. 201192A) and Fix/Perm buffer overnight. Samples were made up with 1:1 ratio of 2x Intercolator-Ir solution and Fix/Perm buffer and incubated for 1 hour at room temperature. Prior to acquisition, cells were washed twice in CSB and once in Maxpar water. Samples were resuspended in 1 mL of Maxpar water and filtered through a 30-µm cell strainer. Cell count was adjusted to 5 × 10^5^ cells/mL containing 10% EQ Four Element Calibration Beads (Standard Biotools, catalog no. 201078). Samples were vortexed well and acquired on Helios mass cytometer (Fluidigm). The samples were normalized using the Fluidigm acquisition software and data were exported as FCS files.

### Mass Cytometry Data Analysis

FCS files were manually processed in FlowJo (RRID:SCR_008520) to gate for live singlets (Supplementary Fig. S1). Processed sample files were analyzed in R using the *CATALYST* (RRID:SCR_017127; ref. 31) package to de-barcode CD45^+^ cells using estimated separation cutoffs according to the barcoding strategy. Individual samples were exported as new FCS files and imported into R using the package *flowCore* (RRID:SCR_002205) for downstream analysis using the CyTOF workflow detailed by Nowicka and colleagues (32). Lineage and functional marker names were appropriately associated with the correct detection channels and diagnostic checks were performed. Cell marker intensities were transformed using an inverse hyperbolic sine (arcsinh) function (cofactor of 5) and scaled between 0 and 1 to give a better representation of the relative differences in expression between cell subsets.

*FlowSOM* (RRID:SCR_016899) and *ConsensusClusterPlus* (RRID:SCR_016954) packages were used to cluster 2000 CD45^+^ cells from each sample into 20 subsets of similarity based on the relative expression of lineage markers (33, 34). The *BuildSOM* function generated a self-organized map (SOM) with 100 nodes from the transformed data. Metaclustering by the *ConsensusClusterPlus* package assigned the SOM nodes to 20 distinct clusters. The data was visualized with t-distributed stochastic neighbor embedding (t-SNE; ref. 35) and heat map (*pheatmap* package) plots that aided with manual merging of the metaclusters into defined immune cell populations. The identified cell populations were visualized and validated to ensure population location and cell marker expression was as expected. Contrasts were made between cell marker expression in the different experimental conditions and manually defined cell populations using *lme4*, *stats*, and *multcomp* R packages. Downstream differential analysis of linear models was performed using *dplyr* on the defined immune cell subsets to investigate cell population abundance and functional marker expression across the different experimental conditions.

### T-Cell Suppression Assays

CD3^+^ T cells were purified from healthy donor human PBMCs using the EasySep Human CD3 Positive Selection Kit II (StemCell Technologies, catalog no. 17851) according to the manufacturer's protocol. Purified CD3^+^ T cells were stained with 5 µmol/L CFSE (Invitrogen, catalog no. C34554) and resuspended in complete RPMI media. CD3^+^ T cells (1 × 10^5^) were cocultured with varying ratios of HNSCC cells and 5 µg/mL anti-CD3 (OKT3, BioLegend) and 10 µg/mL anti-CD28 (CD28.2, BioLegend) stimulation with/without 10 µg/mL CDA in 100 µL in round-bottom 96-well plates. Following a 4-day incubation at 37°C and 5% CO_2_, T cells were stained with APC-conjugated CD8 (SK1, BioLegend) and CD8^+^ T-cell proliferation was assessed by flow cytometry using the Attune NxT Focus cytometer (Thermo Fisher Scientific) and data were analyzed with FlowJo software.

### NK-Cell and Monocyte Degranulation

Healthy donor PBMCs (5 × 10^5^) were plated with HNSCC cells (1 × 10^4^; 50:1 ratio) ± 10 µg/mL CDA in 200 µL total volume in flat-bottom 96-well plates. Ten microliters of PE-conjugated CD107a (H4A3, BioLegend) was added to each well and plates were incubated for a total of 5 hours at 37°C and 5% CO_2_. After 1 hour, 5 µL of a monensin solution [1:37.5 ratio of GolgiStop (BD Biosciences, catalog no. 51–2092KZ) to RPMI complete media] was added to each well. PBMCs were collected following the 5-hour incubation and stained for APC-conjugated CD56 (CMSSB, Thermo Fisher Scientific) and FITC-conjugated CD3 (OKT3, BioLegend). Degranulation was assessed by flow cytometry using the Attune NxT Focus cytometer (Thermo Fisher Scientific) and data were analyzed with FlowJo software.

### Antibody-Dependent Cellular Cytotoxicity Assay

HNSCC cells were stained with 1 µmol/L calcein AM in calcein AM buffer (R&D Systems, catalog no. 4892–010-K) for 30 minutes at 37°C in a 5% CO_2_ humidified incubator. HNSCC cells were washed three times in appropriate cell culture media and plated (1 × 10^4^) in a 96-well flat bottom plate. Healthy donor PBMCs (5 × 10^5^) were added (50:1 ratio) with/without 10 µg/mL CDA, 10 µg/mL cetuximab (SelleckChem, catalog no. A2000, RRID:AB_2893090) or combination treatment in 200 µL total volume. 1% Triton X-100 (Sigma, catalog no. T8532) was used as a positive control for cell lysis. After 5 hours, the plate was centrifuged and 100 µL cell culture supernatant plated in a black-walled 96-well plate for reading on a fluorescent plate reader (485–530 nm).

### IHC

Studies were conducted in accordance with the Declaration of Helsinki. OPSCC primary tissue samples were obtained from individuals undergoing surgery as a primary treatment by Liverpool University Biobank following written informed consent under the NHS Research Ethics Committee (REC) 10/H1002/53 and REC 10/H1010/37. Sections from oropharyngeal-derived tissue microarrays (TMA) from 170 patients with HPV^−^ and HPV^+^ HNSCC (Table 1) were processed and stained using the Bond RX^m^ autostainer (Leica). Sections were stained using anti-rabbit STING (1:400, Cell Signaling Technology) or anti-rabbit IFNβ (1:50, Invitrogen) before HRP-linking for DAB detection and hematoxylin counterstaining. Aperio slide scanner (Leica) was used to scan mounted TMA slides at 40× magnification. STING and IFNβ staining intensity was evaluated using QuPath software (RRID:SCR_018257) that generated Histo-scores (H-scores; ref. 36) from cytoplasmic or total cell DAB optical density measurements respectively of each tumor or immune cell within each sample. H-scores were averaged between the 2/3 samples available for each patient and provided a quantitative measure of STING and IFNβ staining levels.

**TABLE 1:** Patient characteristics of the OPSCC TMA cohort

	Total oropharyngeal samples, *n* (%)		Tumor core	Tumor AF	Normal
HPV status					
HPV^−^	61	37%	51	44	35
HPV^+^	102	63%	62	48	31
Unknown	7		3	4	5
Gender					
Female	19	22%	17	15	9
Male	66	78%	46	40	20
Unknown	85		52	40	41
Age at presentation					
(Data available for HPV-samples only)					
<60 years	18	50%	17	18	9
>60 years	18	50%	18	16	11
Unknown	134		193	112	52
Tumor site					
Base of tongue	39	23%	24	21	13
Oropharynx	12	7%	10	8	7
Soft palate	15	9%	10	9	7
Tonsil	101	61%	70	56	41
Unknown	3				
Recurrence					
No	96	77%	61	52	34
Yes	28	23%	20	16	15
Unknown	46		36	29	22
T stage					
T1	14	10%	10	5	3
T2	78	52%	52	54	34
T3	48	32%	31	22	16
T4	9	6%	9	6	3
Unknown	21		13	8	13
N stage					
N0	27	17%	22	16	14
N1	24	16%	17	12	8
N2	104	67%	66	59	37
Unknown	15		11	9	12
Survival (≥5 years)					
Alive	88	58%	65	53	33
Deceased	64	42%	38	32	28
Unknown	18		13	11	10

### Statistical Analysis

All data were presented as mean ± SD and statistical analyses were performed using GraphPad Prism software v.8 GraphPad Prism (RRID:SCR_002798). Statistical analyses between multiple groups were performed using one-way ANOVA or two-way ANOVA with Dunnett multiple comparisons test as needed. Comparisons between T-cell proliferation, monocyte, and NK-cell degranulation, and TMA analyses were performed using Student *t* test. Survival curves were constructed using the Kaplan–Meier method and compared using the log-rank (Mantel–Cox) test. For all analyses ***, *P* < 0.001; **, *P* < 0.01; *, *P* < 0.05.

### Data Availability Statement

The data generated in this study are available upon request from the corresponding author. The RNA-sequencing data generated for this study is publicly available in Gene Expression Omnibus through the accession number GSE250305.

## Results

### STING Expression in HNSCC Cell Lines is Dependent on HPV Status

**FIGURE 1:**
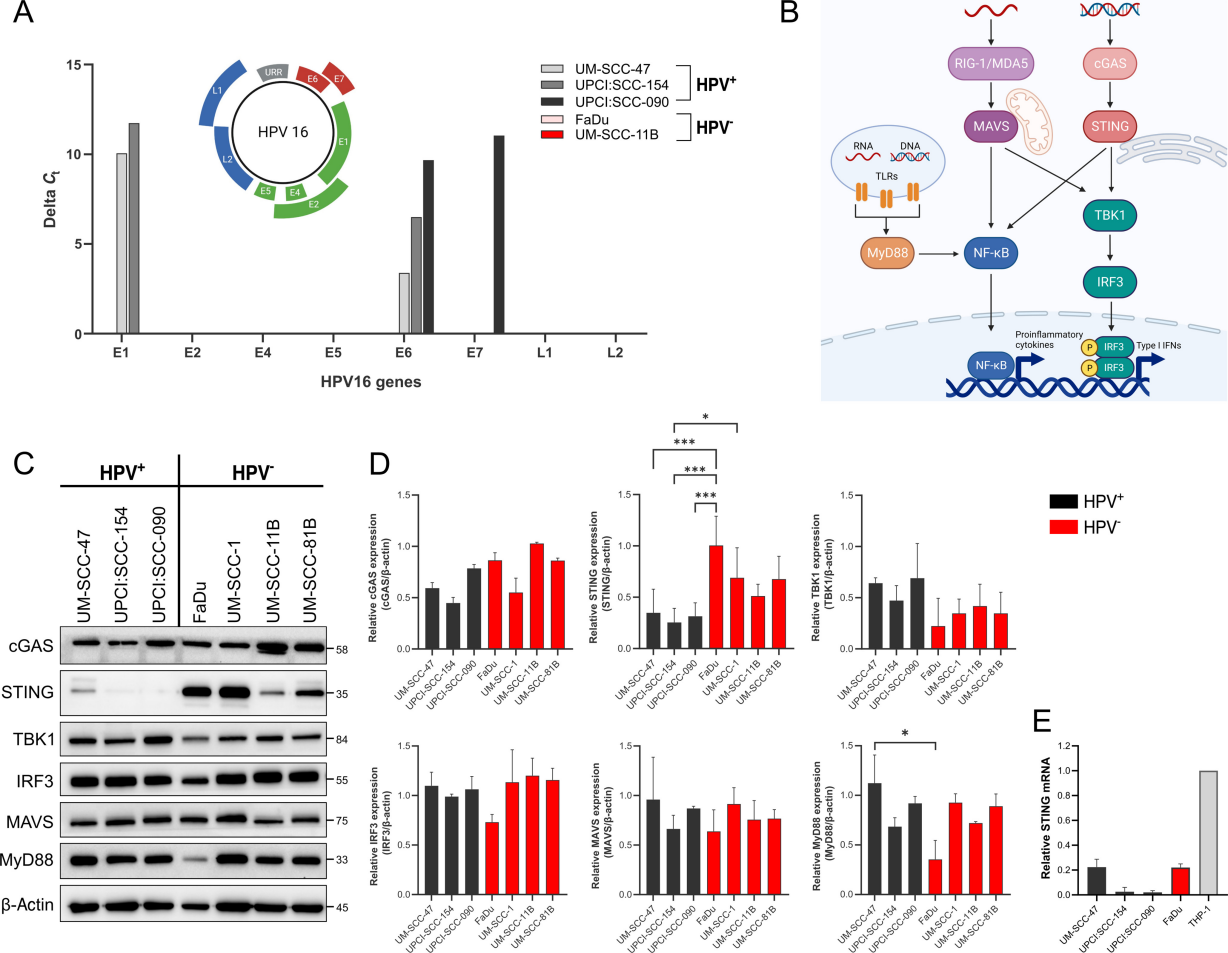
STING is differentially expressed in HPV^+^ and HPV^−^ HNSCC cell lines. **A,** RT-qPCR analysis of the 8 genes of the HPV16 genome (as shown in the schema) in HNSCC cell lines. **B,** Overview of innate immune DNA/RNA sensing mechanisms and their associated signaling components. **C,** Proteins were extracted from HPV^+^ and HPV^−^ HNSCC cell lines and immunoblots probed with antibodies against cGAS, STING, TBK1, IRF3, MAVS, MyD88 and β-actin. **D,** Quantitative analysis of proteins probed in C normalized to β-actin (*n* = 3). **E,** RT-qPCR analysis of basal STING expression in HNSCC cells. Gene expression was normalized to β-actin and THP-1 expression used as a reference to calculate 2^−ΔΔ^*^C^*^t^ values (*n* = 2). Each value represents mean ± SD. *, *P* < 0.05; ***, *P* < 0.001.

Experiments were conducted using seven commercially available and established HNSCC cell lines, including four from HPV^−^ and three from HPV^+^ phenotypic origins. We first confirmed the genomic integration of essential HPV16 genes in the HPV^+^ cell lines by RT-qPCR. (Fig. 1A). All HPV^+^ characterized cell lines expressed E6, with UM-SCC-47 and UPCI:SCC-154 also transcribing E1 and UPCI:SCC-090 expressing E7. The HPV^−^ cell lines investigated did not express any of the HPV genes. This provided assurance that HPV^+^ HNSCC cells constitutively express HPV genes necessary for viral replication and comparisons can be made between the characterized cell lines based on HPV phenotype. After validation of HPV16 genomic integration in the cell lines, we characterized the basal protein expression of STING and associated pathway components in HNSCC cell lines (Fig. 1B). STING showed varied expression across the different HNSCC cell lines, with HPV^−^ cells displaying higher expression compared with low or absent levels in the HPV^+^ cell lines (Fig. 1C and D). Upstream cGAS and downstream TBK1 and IRF3 exhibited similar levels of expression across all HNSCC cell lines regardless of HPV status. Mitochondrial antiviral-signaling protein (MAVS) and myeloid differentiation primary response 88 (MyD88) are two other adaptor proteins that play key roles in antiviral innate immune responses via retinoic acid-inducible gene I- (RIG-I) and toll-like receptor- (TLR)-mediated activation respectively (37–39). Both MAVS and MyD88 demonstrated more consistent and strong expression across all HNSCC cell lines, with only HPV^−^ FaDu showing diminished expression of MyD88 compared with HPV^+^ UM-SCC-47 (Fig. 1C and D). RT-qPCR analysis (Fig. 1E) highlighted that STING is transcribed at markedly diminished levels compared with THP-1 cells (positive control cell line) in cells derived from both HPV^−^ and HPV^+^ HNSCCs. In particular, levels of STING were near absent in the HPV^+^ cell lines. STING was the only of these DNA sensors characterized to show differential expression across the cell lines dependent on HPV status, suggesting its dysregulation may play a role in producing the unique properties of HPV^+^ and HPV^−^ HNSCC phenotypes.

### STING Pathway Activation is Dysfunctional in HNSCC Cell Lines

**FIGURE 2:**
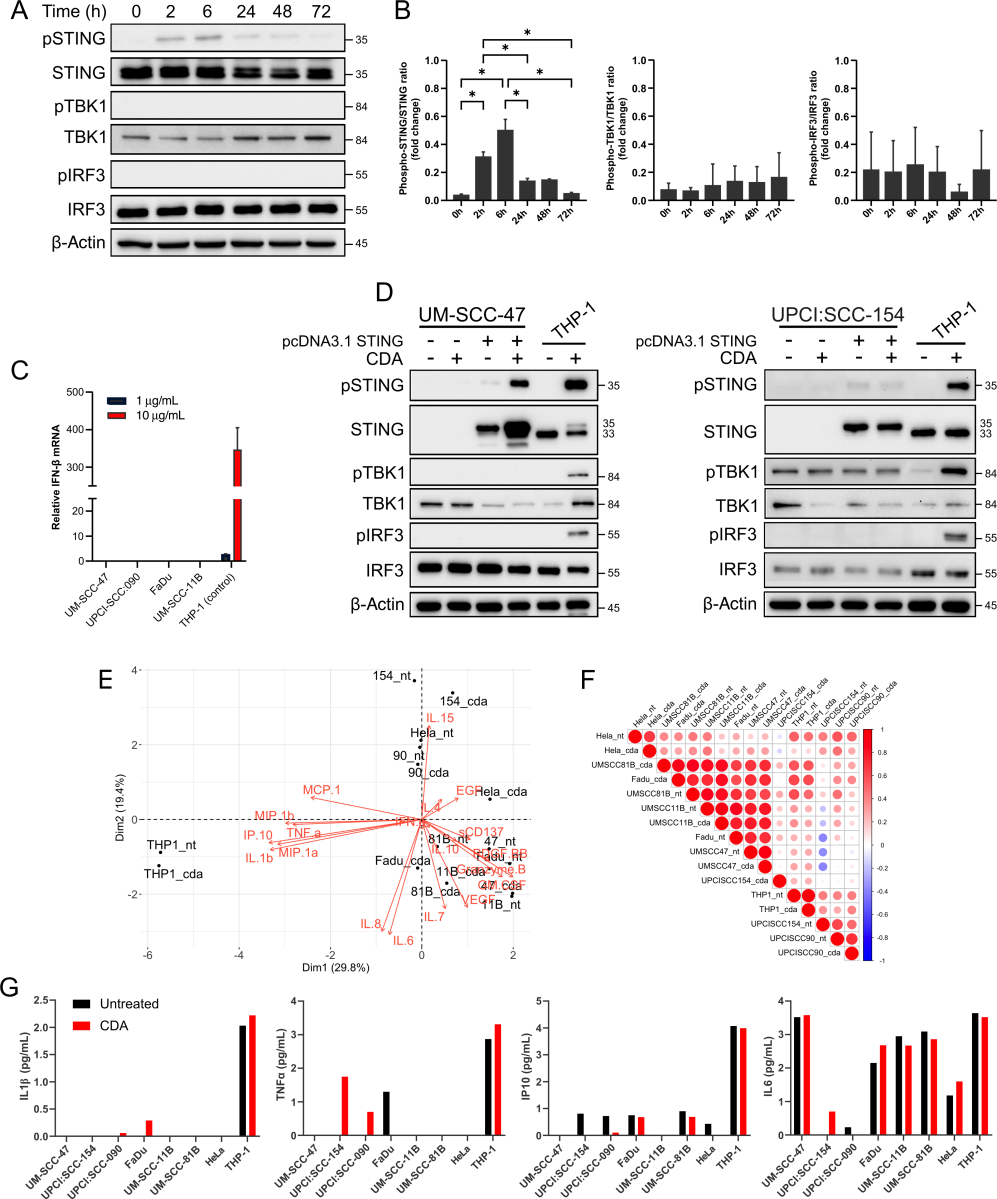
STING pathway signaling is absent in HSNCC cell lines. **A,** FaDu cells were stimulated with 10 µg/mL ML RR-S2 CDA (CDA) over a period of 72 hours and proteins were extracted for Western blot analysis of STING pathway activation. **B,** Densitometry plots represent the relative levels of phosphorylated proteins normalized against total protein expression. Mean ± SD (*n* = 3). *, *P* < 0.05. **C,** RT-qPCR analysis of IFNβ induction in HNSCC cell lines following 6-hour treatment with 1 or 10 µg/mL CDA (*n* = 2). **D,** HPV^+^ UM-SCC-47 and UPCI:SCC-154 cells were transiently transfected with STING for 72 hours. Cells were treated with 10 µg/mL CDA for the final 6 hours and proteins extracted for immunoblot analysis of STING pathway activation (*n* = 2). THP-1 cells were included as a positive control. PCA biplot (**E**) and correlation matrix (**F**) of innate immune cytokine release from HNSCC cells (THP-1 and HeLa controls) ± 10 µg/mL CDA stimulation for 6 hours measured by multiplex ELISA. Color scale represents Pearson correlation coefficient. **G,** Bar charts depicting ELISA measurement of IL1β, TNFα, IP-10, and IL6 levels of secretion from the cells (*n* = 3).

We next investigated the impact of STING activation on downstream pathway signaling in the HPV^−^ FaDu cell line, as it was found to have the highest basal expression of STING (Fig. 1C and D). Cells were treated with 10 µg/mL of the STING agonist ML RR-S2 CDA (CDA) over a 72-hour time course and protein expression was analyzed by Western blot (Fig. 2A). CDA treatment led to phosphorylation of STING at 2- and 6-hour time points, but this did not lead to downstream phosphorylation of TBK1 or IRF3 (Fig. 2A and B). RT-qPCR analysis following treatment with either 1 or 10 µg/mL of CDA showed no induction of IFNβ transcription in any of the HNSCC cell lines assessed (Fig. 2C). STING-mediated signaling and subsequent type-I IFN induction was absent across all HNSCC cell lines assessed. Furthermore, stimulation with CDA or 2′3′-cGAMP did not alter the expression of surface markers (CD47, EGFR, PD-L1, CD80, HLA-A2 and HLA-DR) on HNSCC cell lines (Supplementary Fig. S2), showing a gross defect in the activation of this pathway. There is recent evidence of the HPV16-mediated degradation of STING as a potential immune evasion tactic utilized by HPV^+^ HNSCC cell lines (40, 41). To investigate whether STING activation could be restored in HPV^+^ HNSCC cells with abundant STING expression, we overexpressed STING in UM-SCC-47 and UPCI:SCC-154 cells and stimulated them with CDA (Fig. 2D). STING was strongly detected in both HPV^+^ cell lines transfected with STING compared with wild-type cells, and stimulation of STING overexpressing UM-SCC-47 cells with CDA led to potent STING phosphorylation, but not phosphorylation of downstream TBK1 and IRF3. STING-expressing UPCI:SCC-154 cells did not show any activation of STING or its downstream components following CDA treatment (Fig. 2D). We used THP-1 cells as a positive control in these experiments as CDA treatment led to potent phosphorylation of STING, TBK1, IRF3, and induction of type-I IFN (Fig 2C and D).

To further explore STING signaling responses, HNSCC cells and control cell lines THP-1 (intact STING signaling) and HeLa (HPV^+^ cervical cell line) were treated with 10 µg/mL CDA for 6 hours and cell supernatants were collected for multiplex ELISA of innate immune cytokines. Principal component analysis (PCA) and correlation matrix of cytokine release highlighted that the cell lines clustered into three groups; THP-1 samples, HPV^−^ samples and HPV^+^ samples (Fig. 2E and F). The only exception was HPV^+^ UM-SCC-47, which clustered more toward the HPV^−^ cell lines, and this was the only HPV^+^ cell line to have detectable STING expression (Fig. 1B–D). Interestingly, HeLa cells, that have integrated HPV18, clustered most closely with the HPV^+^ HNSCC samples, suggesting that there may be a universal innate immune targeting in HPV cancers that is driven by the virus itself. THP-1 cells, used as positive control, were the only cell lines to secrete proinflammatory cytokines IL1β, TNFα, and IP-10 (Fig. 2E–G). There was more universal expression of NFκB-inducible IL6 (Fig. 2G) across all the cell lines investigated, but this was not necessarily induced by drug treatments. These results further indicate that STING-TBK1-IRF3–mediated activation is blunted in HNSCC cells, but suggest that STING-IKK-NF-κB axis may still be functional.

### Differential Gene Expression in HPV^+^ and HPV^−^ HNSCCs

**FIGURE 3:**
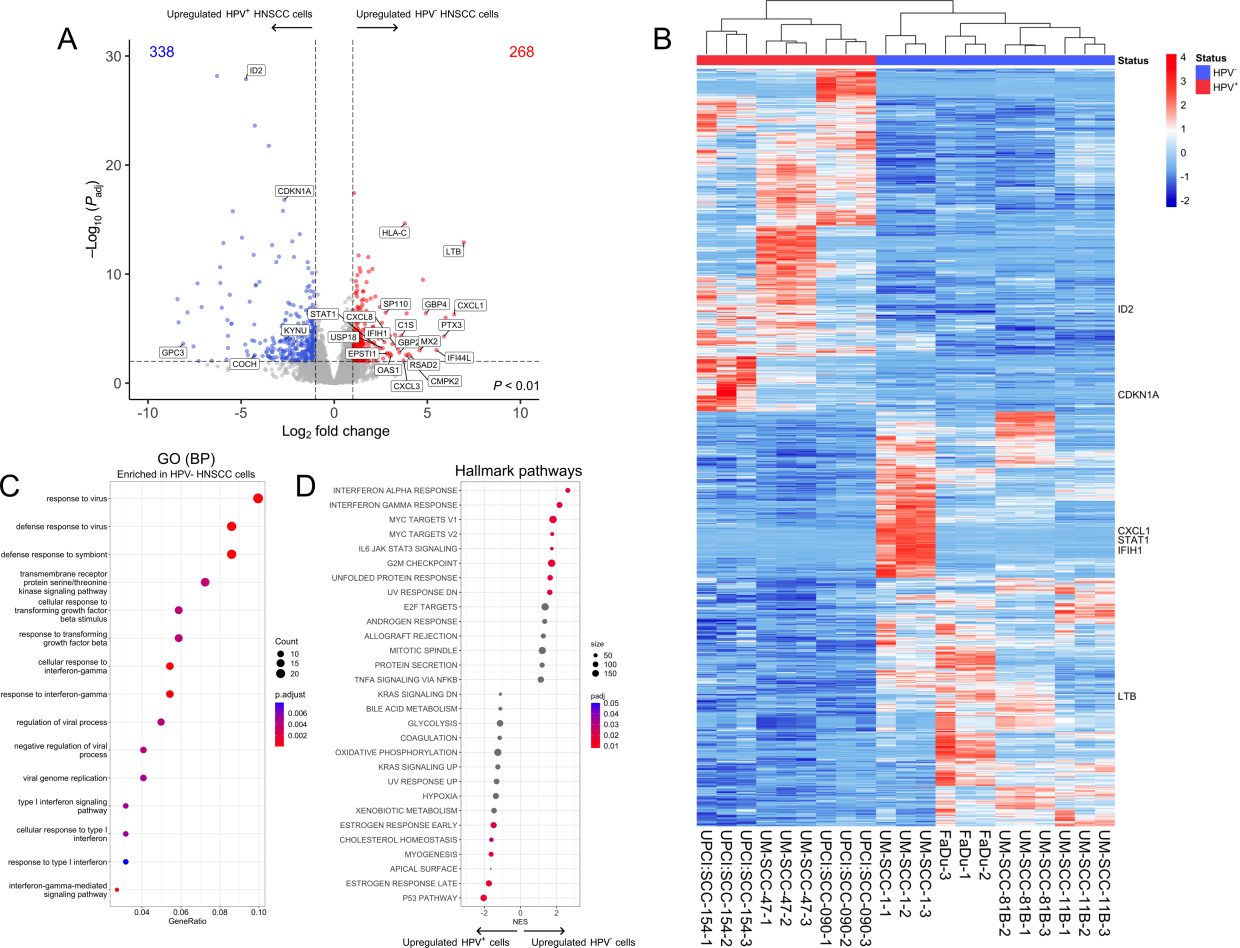
Analysis of differentially expressed genes in HPV^−^ and HPV^+^ HNSCC cells. Total RNA was harvested from wild-type HNSCC cells of both HPV^−^ (4 cell lines) and HPV^+^ (3 cell lines) phenotypic origins and mRNA transcript levels were determined. Gene expression was normalized, and gene values averaged and log-transformed. Differential expression was investigated using an R-based workflow with the *DeSeq2* package. **A,** Volcano plot showing differentially expressed genes between HNSCC cells dependent on HPV phenotype. Red and blue dots indicate significantly upregulated genes in HPV^−^ and HPV^+^ HNSCC cell lines respectively. Significance defined as 2-fold change and adjusted *P* < 0.01, significant inflammatory pathway genes highlighted. **B,** Heat map of significantly differentially expressed genes between HPV^−^ and HPV^+^ HNSCC cell lines. **C,** Gene ontology (GO) analysis of biological process (BP) categories enriched in HPV^−^ cell lines (top 15 pathways shown). **D,** Gene-set enrichment analysis of Hallmark pathways differentially expressed between HNSCC HPV phenotypes, ranked by normalized enrichment score (NES). Positive NES, upregulated in HPV^−^ cells; negative NES, upregulated in HPV^+^ cells, color indicates significance. Adjusted *P* value of each process/pathway indicated by relevant key (*n* = 3 biological replicates).

HPV^+^ and HPV^−^ HNSCCs are defined as two etiologically distinct diseases that are driven by unique risk factors (42), and the results above demonstrate they possess distinct antiviral sensing mechanisms. We further interrogated their transcriptional profiles to unravel differences in innate immune-sensing mechanisms based on HPV phenotypes by next-generation sequencing. Initial analysis determined 338 and 268 genes were upregulated in HPV^+^ and HPV^−^ HNSCC cell lines respectively (Fig. 3A and B; and Supplementary Data S1), highlighting the heterogeneity driven by the absence or presence of HPV. The three biological replicates collected from each HNSCC cell lines clustered closely together on a PCA plot, but cell lines did not cluster based on HPV status (Supplementary Fig. S3), suggesting transcriptional heterogeneity across all cell lines assessed. Genes associated with type-I IFN signaling, *IRF7* and *STAT1*, were upregulated in HPV^−^ cells and gene ontology (GO) analysis of biological processes found defense responses against viruses and general immune responses were significantly enhanced in HPV^−^ HNSCC cells (Fig. 3C). Further analysis of transcriptional differences in hallmark pathways demonstrated that HPV^−^ cells have significantly higher expression of genes involved in responses to IFNα, responses to IFNγ, and enhanced IL6-JAK-STAT3 signaling compared with HPV^+^ cells (Fig. 3D). When we assessed response to IFNγ in cell lines by Western blotting, we found that levels of STAT1 were increased in HPV^−^ cell lines following treatment with IFNγ, while phosphorylation of STAT1 was most pronounced in HPV^−^ cell line UMSCC-81B (Supplementary Fig. S4). Overall, this suggests that HPV^+^ HNSCC cells have suppressed immune responses and downregulation of STING protein expression may be a result of or involved in producing these effects.

### PBMC Responses to HNSCC Cells Following STING Activation

**FIGURE 4:**
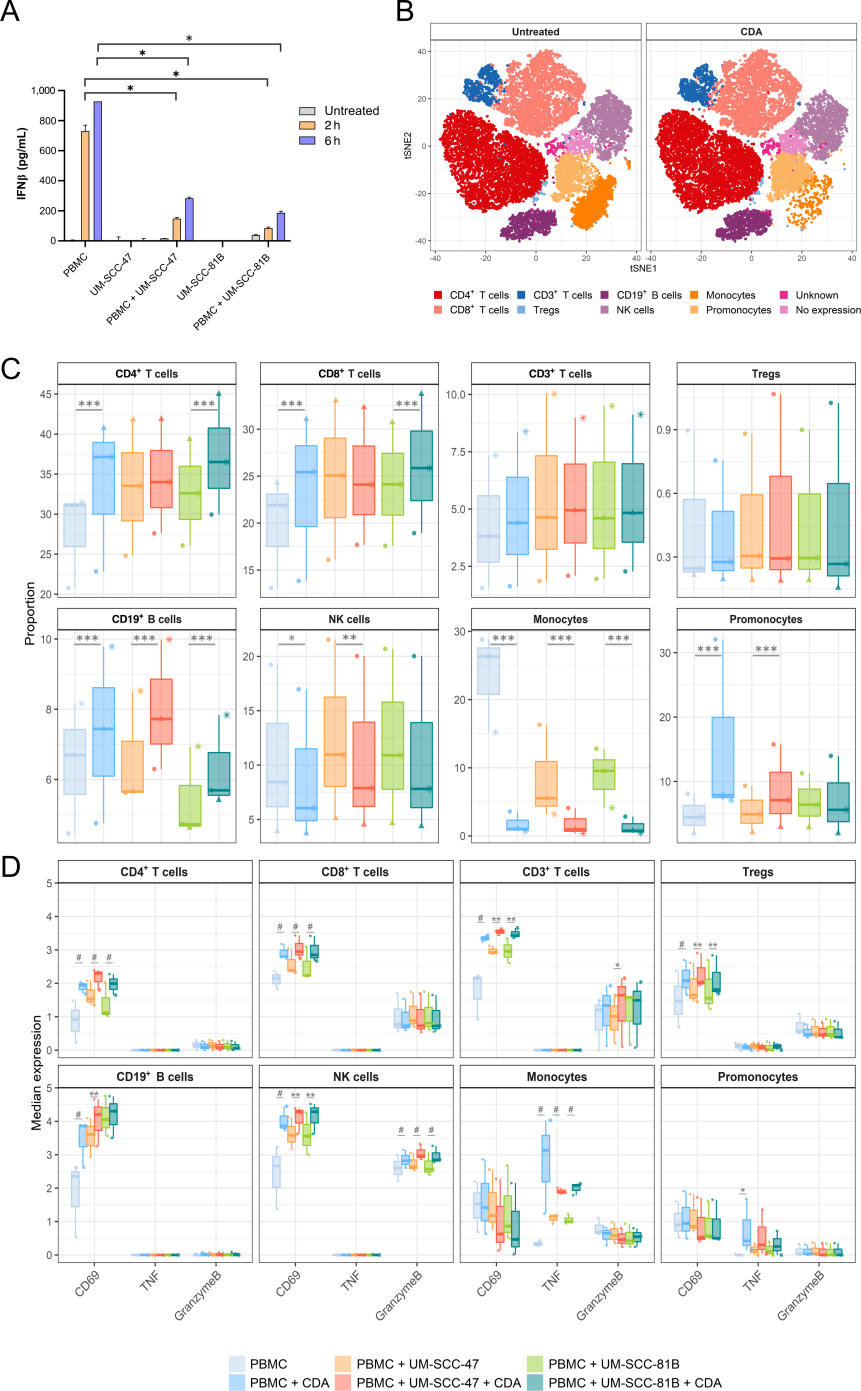
PBMC responses against HNSCC cells following STING stimulation. Healthy donor PBMCs were cocultured with HPV^+^ UM-SCC-47 or HPV^−^ UM-SCC-81B cells alongside for 2 or 6 hours at 10 µg/mL ML RR-S2 CDA (CDA) stimulation before (**A**) secreted IFNβ was measured by ELISA (*n* = 4) or isolation of PBMCs for mass cytometry analysis (*n* = 3). **B,** t-SNE plots depict general changes in immune cell proportions observed across all untreated and CDA-stimulated PBMC conditions. Boxplots show changes in immune cell proportions (**C**) and their activation (CD69), proinflammatory (TNF), and functional markers (Granzyme B) across each condition **D**. *** and ^#^, *P* < 0.001; **, *P* < 0.01; *, *P* < 0.05.

Antitumor immune responses following STING agonist treatment have been shown to rely on robust immune cell activation (22, 23). Although absent or dysfunctional in tumor cells, we hypothesise that the adjuvant properties of STING agonists can be exploited via activation of immune cells. STING expression has been reported in human T cells (43), monocytes, macrophages (20), and NK cells (44). We therefore investigated immune cell responses against HNSCC cells following STING activation. First, we cocultured healthy donor PBMCs with UM-SCC-47 (HPV^+^) or UM-SCC-81B (HPV^−^) cells for 2 or 6 hours in the presence of CDA and collected the cell culture supernatant to measure the levels of IFNβ secretion by ELISA. PBMCs alone secreted high levels of IFNβ following STING stimulation, but the addition of either UM-SCC-47 or UM-SCC-81B cells significantly diminished PBMC-mediated IFNβ production (Fig. 4A), a phenomenon not observed when cells were cocultured in transwell (Supplementary Fig. S5A), showing that the inhibition of IFNβ is contact dependent. However, other proinflammatory cytokines TNFα, IL1β and IL6, primarily driven by STING-mediated inflammasome and NF-κB activation, were relatively unaffected (Supplementary Fig. S5B). To better understand the effects CDA stimulation has on immune cells in cocultures with HNSCC cells, we repeated the experiment using 10 µg/mL CDA stimulation for 6 hours and collected the PBMCs for mass cytometry analysis. During this period of coculture, we observed similar viability across the cell lines tested in the presence of CDA, and when cocultured with PBMCs (Supplementary Fig. S6). Eight immune cell populations were identified using unsupervised clustering and manual merging (Supplementary Fig. S7) and changes in immune cell proportions were comparable across all untreated and CDA-stimulated conditions (Fig. 4B). Addition of the STING agonist significantly increased the proportion of T cells, B cells, and promonocytes, reduced NK cells, and diminished the monocyte population (Fig. 4C). Flow cytometry analysis of PBMCs stained with Annexin V and propidium iodide (PI) showed that treatment with 10 µg/mL CDA for 6 hours did not lead to the cell death of lymphocytes, but reduction in monocyte population confirming the results seen with mass cytometry analysis (Supplementary Fig. S8). We further looked at expression of proinflammatory markers in the immune cell subsets collected from each condition. There was increased activation of several immune cell subsets within all CDA-stimulated conditions compared with their untreated counterpart (Fig. 4D). The early activation marker CD69 was significantly upregulated on all T-cell subsets, B cells, and NK cells. NK cells had increased levels of granzyme B, indicating STING activation may enhance NK-cell lytic ability. TNF, a key promoter of inflammation, was produced in large quantities by the monocyte population following STING activation. In addition, HNSCC and PBMC cocultures had increased levels of proinflammatory markers compared with PBMCs alone. This indicates that PBMCs are activated in the presence of HNSCC cells and the addition of STING stimulation further amplifies these proinflammatory responses independently of type-I IFNs.

### Immune Cell Functional Responses to HNSCC Cells Following STING Activation

**FIGURE 5:**
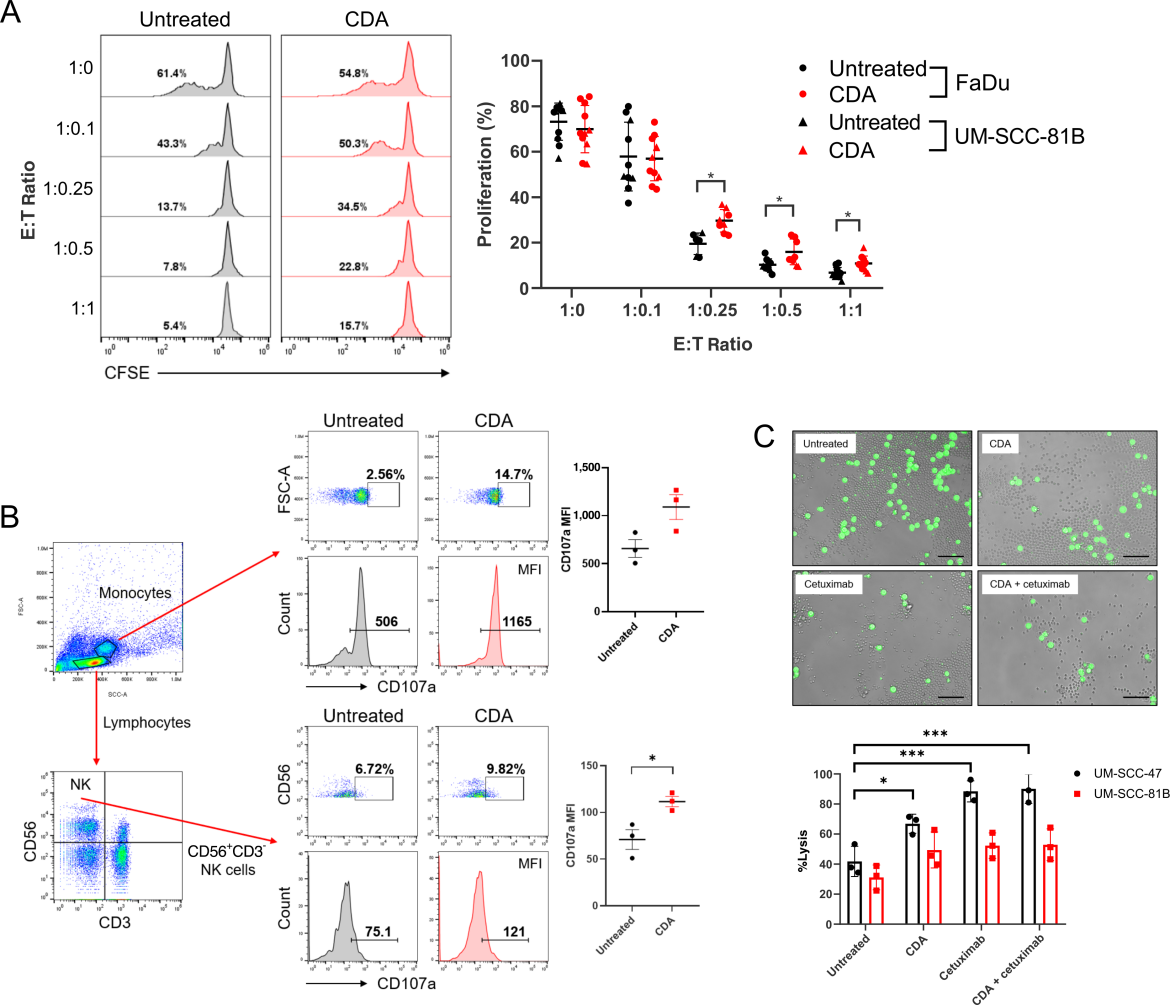
STING activation enhances CD8^+^ T cell, NK cell, and monocyte function against HNSCC. CFSE-labeled CD3^+^ T cells stimulated with anti-CD3 and anti-CD28 mAbs were cocultured with varying ratios of FaDu or UM-SCC-81B cells ± 10 µg/mL CDA stimulation for 4 days. **A,** Representative flow cytometry histograms of CD8^+^ T-cell proliferation against FaDu cells. Numbers in histograms depict % proliferation. Adjacent graph shows mean % proliferation ± SD at each E:T ratio (T cell:HNSCC). PBMCs were cocultured with FaDu cells (50:1 ratio) for 5 hours ± 10 µg/mL CDA stimulation. **B,** Representative flow cytometry plots and histograms of CD107a expression in monocyte and NK-cell (CD56^+^CD3^−^) populations. Numbers in plots and histograms depict % CD107a^+^ cells and CD107a MFI, respectively. Accompanying graphs show mean CD107a MFI ± SD for each condition. PBMCs were cocultured with calcein AM–stained HNSCC cells (50:1 ratio) for 6 hours ± 10 µg/mL CDA, 10 µg/mL cetuximab, or combination treatment. **C,** Representative images of UM-SCC-47 cells (live cells stained green by calcein AM) following 6-hour cocultures and quantitative analysis of target cell lysis measured as calcein AM release in the supernatant (mean ± SD). For all panels, *n* ≥ 3 biological replicates. ***, *P* < 0.001; **, *P* < 0.01; *, *P* < 0.05.

We next analyzed T-cell proliferation, NK-cell cytotoxicity, and monocytic degranulation in response to HNSCC cells in the presence of STING stimulation. Recent studies demonstrate that STING activation can mediate priming of CD8^+^ T cells in tumor-bearing mice (22, 23). To determine whether the same effects on T-cell proliferation are observed in humans, *in vitro* CD3^+^ T-cell and HNSCC cocultures were set up for 4 days ± 10 µg/mL CDA stimulation. Increasing numbers of FaDu or UM-SCC-81B cells in the cocultures led to suppression of CD8^+^ T cells (Fig. 5A and B). However, the addition of CDA reversed the suppressive effect of HNSCC cells on CD8^+^ T-cell proliferation at the higher effector-to-target ratios (1:0.25, 1:0.5, 1:1). This suggests that treatment with STING agonists may enhance T-cell proliferation within the TME of larger and intact HNSCC tumors and may aid in promoting antitumor targeted responses.

Tumor cell lysis via release of cytotoxic granules is a key antitumoral property of NK cells (45) and higher infiltration of NK cells in HNSCC TMEs is associated with improved survival (46). We investigated NK cell–mediated degranulation against HNSCC cells with or without CDA stimulation. STING agonist treatment significantly enhanced levels of the degranulation marker CD107a in the NK cells (Fig. 5B) and suggests NK-cell cytotoxicity against HNSCC cells is greater with STING activation. Interestingly, when we evaluated CD107a-mediated degranulation in the monocyte population, we also saw very high levels of degranulation against HNSCC cells (Fig. 5B). In addition, NK cell–mediated ADCC is also exploited in the treatment of various cancers, and is enhanced in patients with HNSCC after treatment with cetuximab, the only clinically approved EGFR-targeted therapy (47). We therefore investigated whether STING activation led to enhanced NK cell–mediated ADCC of target HNSCC cells in the presence of cetuximab. EGFR expression was variable across the HNSCC cell lines. We chose UM-SCC-47 (highest levels of EGFR) and UM-SCC-81B (medium levels of EGFR expression) for evaluating ADCC (Supplementary Fig. S9). NK-cell ADCC was significantly enhanced against UM-SCC-47 cells following CDA, cetuximab, and CDA + cetuximab combination treatments compared with the untreated control (Fig. 5C). Cetuximab and CDA + cetuximab combination treatment had comparable levels of ADCC activation, suggesting cetuximab is the main driver of activation. This effect was not observed against HPV^−^ UM-SCC-81B cells, indicating that high levels of EGFR expression on HNSCC cells is vital for effective NK cell–mediated ADCC with the anti-EGFR antibody cetuximab. However, tumor cell lysis was still significantly enhanced against UM-SCC-47 cells following STING activation in the absence of cetuximab, aligning with recent studies suggesting STING may play an important role in NK-cell activation and antitumor effects (48). Overall, STING stimulation enhances both innate and adaptive immune cell responses against HNSCC cells and STING activation in immune cells, independent of their expression/activation in tumor cells may represent an important strategy to improve antitumor responses against HNSCC cells.

### STING Expression in HNSCC Primary Tumor Samples

Following studies using HNSCC cell lines and coculture systems, we assessed STING protein expression in primary human clinical samples. We have previously analyzed the TCGA dataset and found STING mRNA was significantly upregulated in HPV^+^ HNSCC samples and those derived from the oropharynx (42). These studies contradict our findings in the HNSCC cell lines, so to further validate primary tissue STING expression we used IHC to investigate STING protein expression in oropharyngeal-derived tissue microarrays (TMA) from 170 patients with HPV^−^ and HPV^+^ HNSCC (Table 1). The STING antibody was initially validated by Western blot and IHC in the FaDu cell line with siRNA-mediated knockdown of STING (Supplementary Fig. S10).

**FIGURE 6:**
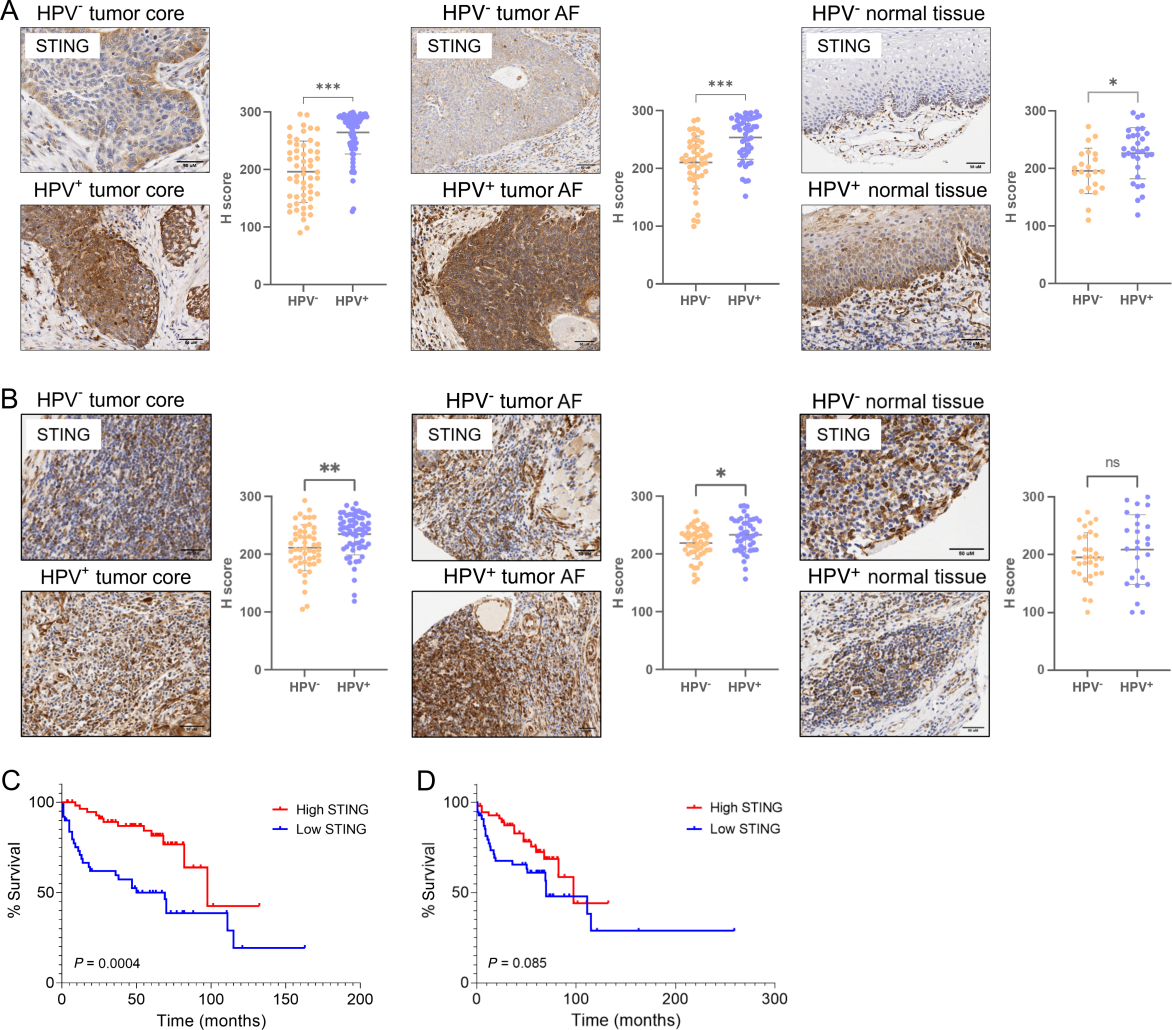
STING expression is upregulated in HPV^+^ HNSCC primary tissue samples. Representative IHC images of HPV^−^ and HPV^+^ tumor cells (**A**) and immune cells (**B**) from oropharyngeal primary tumor core, tumor advancing front (AF), and adjacent normal tissue samples stained with an antibody against STING and counterstained with hematoxylin. Scale bars, 50 µm. Accompanying dot plots depict STING relative staining intensity (H-score). High and low STING expression was stratified by the median H-score value (245 for tumor cells, 229 for immune cells). Kaplan–Meier curves stratifying patient overall survival by STING H-score expression in tumor cells (**C**) and immune cells (**D**) found in tumour core samples (log-rank test). ***, *P* < 0.001; **, *P* < 0.01; *, *P* < 0.05.

The oropharyngeal TMAs were stained for STING and protein expression was compared between HPV^−^ and HPV^+^ patient sections. Across the three tissue types assessed; tumor core (*P* < 0.001), tumor advancing front (AF; *P* < 0.001), and normal adjacent tissue (*P* < 0.05), STING protein expression was found to be significantly higher in tumor cells (epithelial cells in normal adjacent tissue) derived from HPV^+^ samples compared with HPV^−^ (Fig. 6A). Whole-slide images of the tissue cores selected as representative images further highlight the differences in staining intensity between patients with HPV^−^ and HPV^+^ HNSCC included in the TMA cohort (Supplementary Fig. S11). In addition, STING was evenly expressed across the HPV^+^-stained tumor sections, whereas STING appeared strongest on the tumor periphery of the HPV^−^ samples. We further investigated the staining intensity of STING in the tumor-infiltrating immune cells found within the TMA sections. This showed similar trends in STING expression as the tumor cells, with significantly higher expression in the surrounding immune cells of the HPV^+^ tumor core (*P* < 0.01) and tumor AF (*P* < 0.05) sections compared with the HPV^−^ samples (Fig. 6B). No difference in immune cell STING protein levels was observed between the HPV^+^ and HPV^−^ adjacent normal tissue samples.

Patient survival was compared on the basis of STING expression levels in the tumor cells (Fig. 6C) and immune cells (Fig. 6D) found in the tumor core sections. Median H-score values (245 tumor cell, 229 immune cell) were used to distinguish high and low STING expression (49). Regardless of HPV status, patients with high STING-expressing tumor cells had increased overall survival (Fig. 6C, *P* = 0.0004) compared with those with lower STING expression. Median survival for patients with high STING expression in tumor cells was 97.5 months compared with 50 months for low STING tumors. However, no correlation was seen between overall survival and STING expression in the immune cells (Fig. 6D, *P* = 0.085), suggesting intrinsic tumor expression of STING may be beneficial for antitumor responses (median survival for patients with high STING expression in immune cells = 97.5 months, low STING = 82 months). These results support our previous analysis of TCGA dataset and confirm STING is expressed at higher levels in HPV^+^ HNSCC primary tissue (40) and further define that high tumor levels of STING has positive prognostic outcomes.

### IFNβ Expression in HNSCC Primary Tumor Samples

**FIGURE 7:**
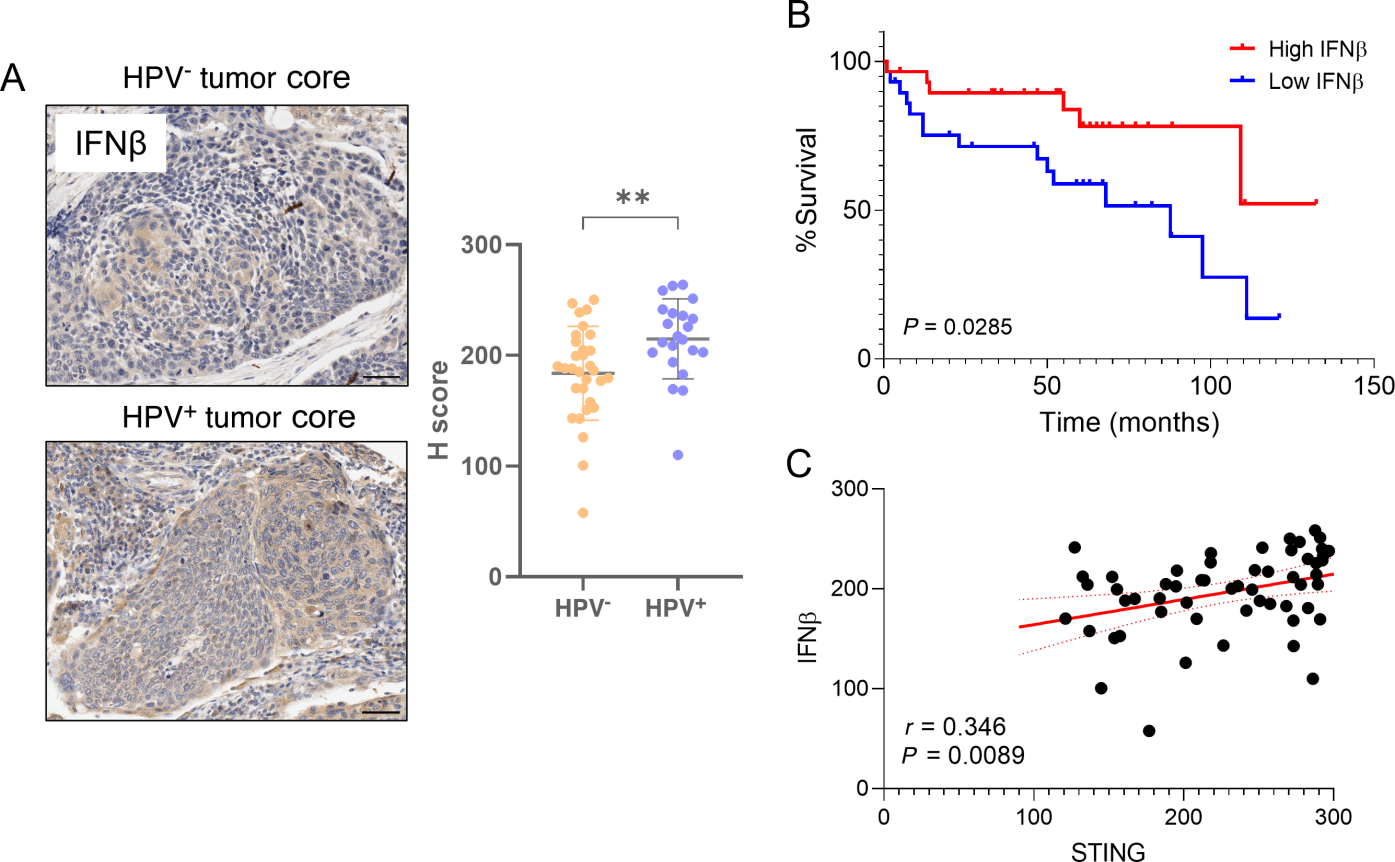
IFNβ expression is upregulated in HPV^+^ HNSCC primary tissue samples and positively correlates with STING expression. **A,** Representative IHC images of HPV^−^ and HPV^+^ tumor cells from oropharyngeal-derived HNSCC primary tumor core samples stained with an antibody against IFNβ and hematoxylin counterstaining. Scale bars, 50 µm. Accompanying dot plots depict the relative staining intensity (H-score) of IFNβ. Error bars = mean ± SD. **B,** Kaplan–Meier curve comparing patient survival by high and low IFNβ expression (stratified by median H-score value of 208) in tumor cells from tumor core sections (log-rank test). **C,** Correlation between tumour cell STING and IFNβ H-scores in tumor core (*r* = 0.346, *P* = 0.0089, *n* = 56) samples of matched OPSCC patients. **, *P* < 0.01.

The observations described above indicate differential expression of STING in HPV^+^ and HPV^−^ HNSCC human patient samples (Fig. 6), with cell lines exhibiting lack of type-I IFN (IFNβ) production in response to STING pathway activation (Fig. 2C). We therefore conducted an IHC analysis for IFNβ expression in the oropharyngeal-derived HNSCC TMAs. Tumor cell IFNβ protein expression was significantly higher in tumor core sections derived from HPV^+^ tissues compared with HPV^−^ tissues (Fig. 7A, *P* < 0.01). Patient survival was compared on the basis of high and low IFNβ expression, stratified by median H-score of 208, in the tumor cells of the tumor core sections. Aligning with the results seen with STING expression and patient survival, patients with higher IFNβ had improved overall survival compared with those with lower IFNβ expression (Fig. 7B, *P* = 0.0285). As IFNβ is an important downstream mediator of STING signaling (50), we compared STING and IFNβ expression in tumor cells from matched patient samples. It was found that STING and IFNβ protein expression were significantly positively correlated (Fig. 7C, *r* = 0.346, *P* = 0.0089), indicating that higher levels of STING in HNSCC tumor cells may contribute to upregulated IFNβ production. Overall, these results indicate that, as opposed to the cell lines, STING pathway activation is intact in HPV^+^ primary human tissues resulting in upregulated innate immune activation compared with HPV^−^ tumors.

## Discussion

The cGAS–STING pathway has been described as one of the major innate immunity pathways that detects tumor-derived DNA and activates antitumoral immune responses. Coadministered with immune checkpoints, cancer vaccines, and adoptive T-cell transfer therapies, synthetic STING agonists are gaining traction as therapeutics that may improve the outcome of cancer treatments. However, systematic structure–function studies of mouse and human STING activation have revealed structural differences and response to ligands (51, 52) and discordance between activation and cell death between human and mouse immune cell populations (53, 54). This may also pose additional challenge in translating STING-activating ligands from mouse studies into human clinical setting. In this study, we provide an extensive characterization of the STING pathway in head and neck cancers using human HNSCC cell lines and primary patient tissues. Present as two distinct phenotypes based on infection with HPV, a DNA virus, we considered head and neck cancers to be an ideal model to study the STING pathway in the context of a virus-associated tumor type.

Our study shows that HPV^+^ HNSCC cell lines expressed undetectable to lower levels of STING protein compared with HPV^−^ cells. This may be due to degradation of STING protein by HPV oncogenes. HPV16 E7 can interact with STING directly via its LXCXE motif (40, 55) or indirectly via a mitochondrial-localized immune system regulator NLRX1 (41) to inhibit or facilitate autophagic degradation of STING respectively. We did not observe diminished expression of other immune sensors MAVS and MyD88 or downstream components of these immune pathways TBK1 and IRF3; suggesting that targeting of STING-specific immune responses may be an important strategy to facilitate tumor progression in HPV^+^ HNSCCs. However, lack of STING downstream signaling in STING-intact HPV^−^ HNSCC cells or STING-overexpressed HPV^+^ cells was unexpected, and suggests that components regulating STING activation may be more complex than previously thought.

Investigation of STING pathway activation in HPV^−^ cell lines found that STING itself was activated by the STING agonist, but downstream induction of IFNβ via TBK1-IRF3 signaling was absent in these cells. Various tumorigenic mechanisms that prevent effective formation of the STING-TBK1-IRF3 signalosome have been described (56, 57), suggesting a similar mechanism may be used by HNSCCs to evade STING-mediated immune responses. Viral targeting of STING-TBK1-IRF3 has been reported (58, 59) for other viruses and may be responsible for the lack of downstream STING responses observed in HPV^+^ HNSCC cell lines following STING overexpression. Classical STING activation also signals through IKK-NF-κB to induce proinflammatory cytokines such as IL6 (20). Our multiplex ELISA results demonstrate that IL6 is robustly expressed across the majority of our HNSCC cell lines, whereas IL1β, TNFα, and IP-10 were only strongly produced by THP-1 cells that potently produce STING-mediated IFNβ. Thus, HNSCC cells may be able to bypass the TBK1-IRF3 route and selectively signal via NF-κB. These proinflammatory mediators may also be able to recruit CXCR3-expressing NK cells and T cells in the TME (60). However, constitutive NF-κB activation is prominent in HNSCCs and thought to be a main contributor in maintaining chronic inflammatory conditions that are widely associated with poor outcomes for patients (61, 62). HNSCC cells may have developed a mechanism to prevent TBK1-IRF3 signaling and manipulate (long-term) STING-IKK-NF-κB activation to help produce a negative inflammatory environment that drives disease.

Other studies have suggested that deficiency of STING in tumor cells may not be the determining factor for generating antitumor responses, and the presence of STING in immune cells may be sufficient to exploit the antitumoral properties of STING agonists (63, 64). In line with this, although downstream STING signaling is lacking in the HNSCC cells themselves, we still observed enhanced immune cell-mediated proinflammatory and cytotoxic responses against HNSCC cells following STING stimulation. Although IFNβ response was blunted in HNSCC-PBMC cocultures in a cell contact manner, other STING-related proinflammatory cytokines were relatively unaffected, showing that the cytotoxic responses can be driven independently of IFNβ. PBMCs had increased levels of proinflammatory markers when cocultured with HNSCC cells, that was further amplified with the addition of STING stimulation. CD8^+^ T-cell proliferation against HNSCC cells was augmented with STING agonist treatment, correlating with expansion of CD8^+^ T cells following STING stimulation in experimental models (22, 23, 65). Addition of STING agonists also enhanced NK-cell and monocyte degranulation against HNSCC cells. Our results agree with studies demonstrating that STING stimulation leads to increased immune cell activation and tumor cell targeting (66), suggesting that STING agonist treatment in addition to conventional therapies would have a positive impact on antitumor immune responses in HNSCCs. In particular, STING agonists may have the greatest impact on HPV^+^ tumors, as they already have elevated infiltration of immune cells, which might assist with current aims to deescalate treatment for patients with HPV^+^ HNSCC.

IHC analysis of oropharyngeal TMAs found STING to be upregulated in HPV^+^ HNSCC primary tissue, correlating with previous studies (42, 67, 68). We saw elevated STING in both the tumor cells and tumor-infiltrating immune cells of HPV^+^ patient tissue, suggesting viral detection is enhanced across a variety of cell types found in the TME. In addition, STING was evenly expressed across the HPV^+^ tumor sections, whereas STING appeared strongest on the tumor periphery of HPV^−^ samples. This may be indicative of different roles STING plays within the two HNSCC phenotypes. Patients with high STING expression in tumor cells had improved overall survival compared with those with lower STING, regardless of tumor HPV status. Previous studies have had contrasting results; with STING expression not correlating with improved outcomes (68) and high RNA and protein STING levels to be associated with significantly better survival (69). Our results indicate that HPV is responsible for upregulated STING protein and IFNβ expression found within HPV^+^ TMEs. This suggests that increased levels of STING and IFNβ may contribute to innate immune activation leading to improved outcomes for patients with HNSCC, correlating with evidence that elevated STING expression enhances responses to radiation treatments (69).

The STING profile observed in HNSCC primary tissue contradicts the higher STING expression observed in HPV^−^ cell lines. It has been widely reported that HPV oncogenes can directly and indirectly inhibit or target STING for degradation (40, 41, 55), indicating why we observed low STING in HPV^+^ HNSCC cell lines. This correlates with the downregulated expression of genes involved in generating immune responses against viruses in HPV^+^ cell lines found in the RNA-sequencing analysis. These HPV-mediated immune evasion strategies have also been reported by other studies (70–72) and the effects of HPV on HNSCC tumor cells may be more pronounced in immortalized cell lines due to long-term exposure to the virus. This effect may further explain why a small subset (∼10%–20%) of HPV^+^ patients have late recurrent disease despite showing good responses to treatment at initial diagnosis (11, 73, 74).

In summary, our comprehensive analysis of the STING pathway in head and neck cancers reveals a fundamental limitation of modeling this disease using HNSCC cell lines to investigate the cGAS-STING pathway. As opposed to primary human HPV^+^ tumors, HPV^+^ HNSCC cell lines do not express the STING protein, while STING-intact HPV^−^ cells have a functional dysregulation of this pathway. This caveat may pose a major obstacle in studies exploring the intrinsic immunogenicity of the HPV virus or the adjuvant properties of STING activation in tumors, which are differentially regulated and activated in cell lines versus primary tumor specimens. However, activation of the STING pathway in immune cell populations potentiates antitumoral effects against HNSCCs, suggesting the use of STING agonists in combination with traditional therapies may have a favorable prognostic outcome.

## Supplementary Material

Supplementary Figure 1 Supplementary Figure 1 shows the gating strategy for mass cytometry data.

Supplementary Figure 2 Supplementary Figure 2 shows changes in cell surface marker expression on HNSCC cells following STING stimulation.

Supplementary Figure 3 Supplementary Figure 3 shows PCA scatter plot of RNA-seq data.

Supplementary Figure 4 Supplementary Figure 4 shows validation of results from RNA-seq analysis.

Supplementary Figure 5 Supplementary Figure 5 demonstrates that HNSCC cells specifically inhibit PBMC-mediated IFNβ production.

Supplementary Figure 6 Supplementary Figure 6 shows the viability of HNSCC cells following STING stimulation and PBMC co-culture.

Supplementary Figure 7 Supplementary Figure 7 shows how immune cell populations were identified from mass cytometry data.

Supplementary Figure 8 Supplementary Figure 8 shows the viability of PBMCs following STING stimulation and HNSCC co-culture.

Supplementary Figure 9 Supplementary Figure 9 shows the surface level expression of EGFR across the HNSCC cell lines.

Supplementary Figure 10 Supplementary Figure 10 provides validation of the STING antibody used for IHC.

Supplementary Figure 11 Supplementary Figure 11 shows the whole slide images of the TMA sections chosen as representative images.

Supplementary Table 1 Supplementary Table 1 shows the antibodies and recombinant proteins used in the study.

Supplementary Table 2 Supplementary Table 2 details the antibodies used for mass cytometry.

Supplementary Data Differentially expressed genes between HPV- and HPV+ HNSCC cell lines using DeSeq2 analysis of RNA-seq data.
