# Supplementary material for: Differential Regulation of the STING Pathway in Human Papillomavirus–Positive and -Negative Head and Neck Cancers
Source: Cancer Res Commun. 2024 Jan 16;4(1):118–33. doi: 10.1158/2767-9764.CRC-23-0299 (PMC10793589; doi:10.1158/2767-9764.CRC-23-0299)
Supplement: Supplementary Figure 1 — shows the gating strategy for mass cytometry data. [file crc-23-0299-s01.pdf]

Supplemental Figure 1

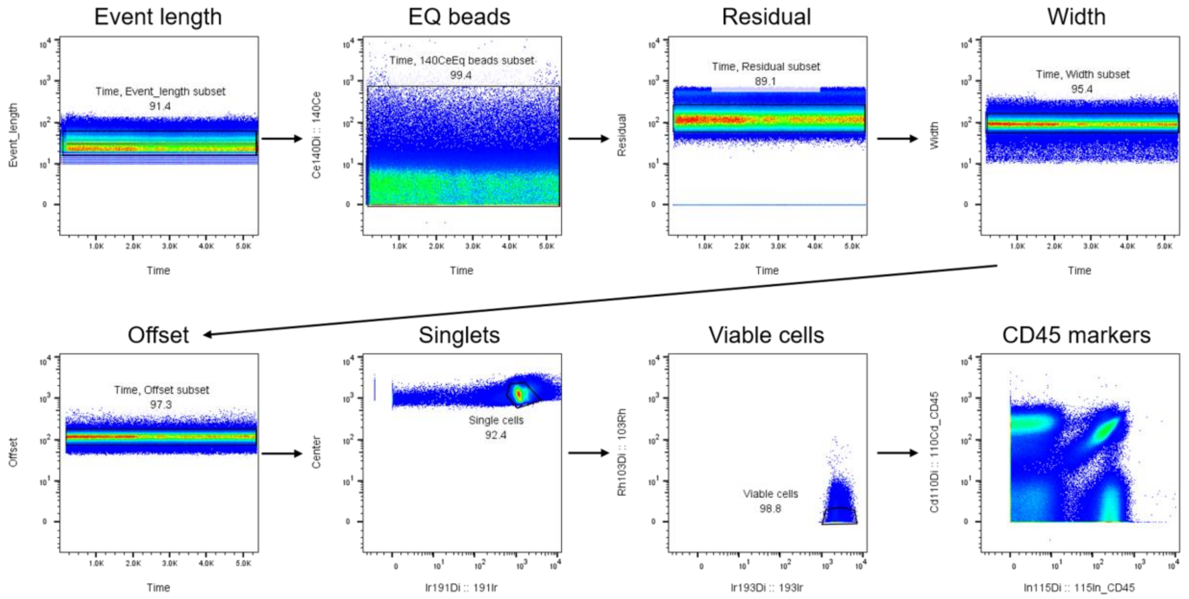

Supplementary figure 1. Gating strategy of mass cytometry data to identify viable single cells for downstream analysis.
