# Supplementary material for: Differential Regulation of the STING Pathway in Human Papillomavirus–Positive and -Negative Head and Neck Cancers
Source: Cancer Res Commun. 2024 Jan 16;4(1):118–33. doi: 10.1158/2767-9764.CRC-23-0299 (PMC10793589; doi:10.1158/2767-9764.CRC-23-0299)
Supplement: Supplementary Figure 2 — shows changes in cell surface marker expression on HNSCC cells following STING stimulation. [file crc-23-0299-s02.pdf]

Supplemental Figure 2

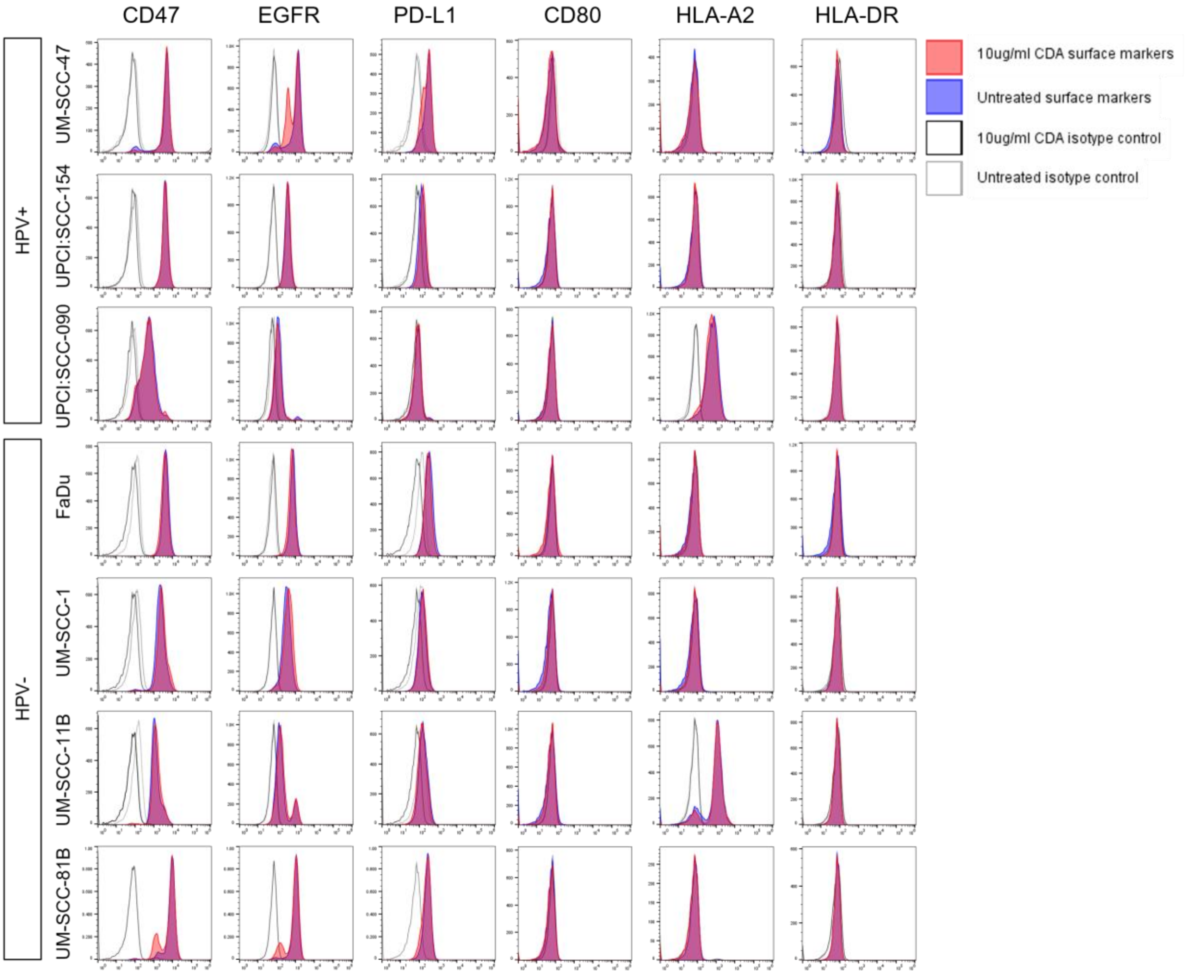

Supplementary figure 2. Cell surface marker changes on HNSCC cells following STING stimulation. HNSCC cell lines were treated +/- 10 µg/mL CDA for 24 h and then stained for cell surface markers CD47, EGFR, PD-L1, CD80, HLA-A2 and HLA-DR for flow cytometry analysis.
