# Supplementary material for: Differential Regulation of the STING Pathway in Human Papillomavirus–Positive and -Negative Head and Neck Cancers
Source: Cancer Res Commun. 2024 Jan 16;4(1):118–33. doi: 10.1158/2767-9764.CRC-23-0299 (PMC10793589; doi:10.1158/2767-9764.CRC-23-0299)
Supplement: Supplementary Figure 3 — shows PCA scatter plot of RNA-seq data. [file crc-23-0299-s03.pdf]

Supplemental Figure 3

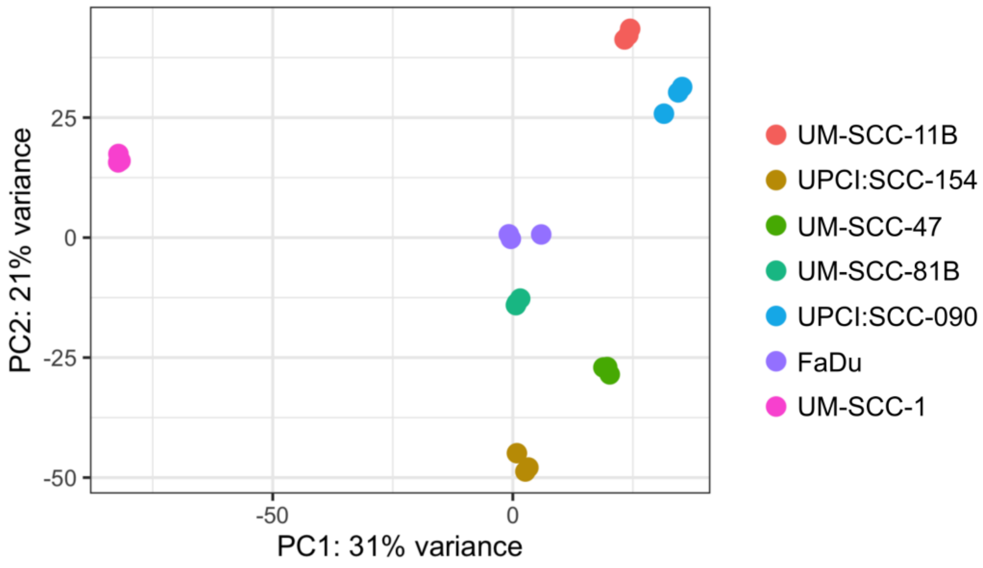

Supplementary figure 3. PCA scatter plot of gene expression in HNSCC cell lines. PCA plot shows variance of three biological replicates of HNSCC cell lines of both HPV^- and HPV^+ origins. The percentage on each axis represent percentage variation.
