# Supplementary material for: Differential Regulation of the STING Pathway in Human Papillomavirus–Positive and -Negative Head and Neck Cancers
Source: Cancer Res Commun. 2024 Jan 16;4(1):118–33. doi: 10.1158/2767-9764.CRC-23-0299 (PMC10793589; doi:10.1158/2767-9764.CRC-23-0299)
Supplement: Supplementary Figure 4 — shows validation of results from RNA-seq analysis. [file crc-23-0299-s04.pdf]

Supplemental Figure 4

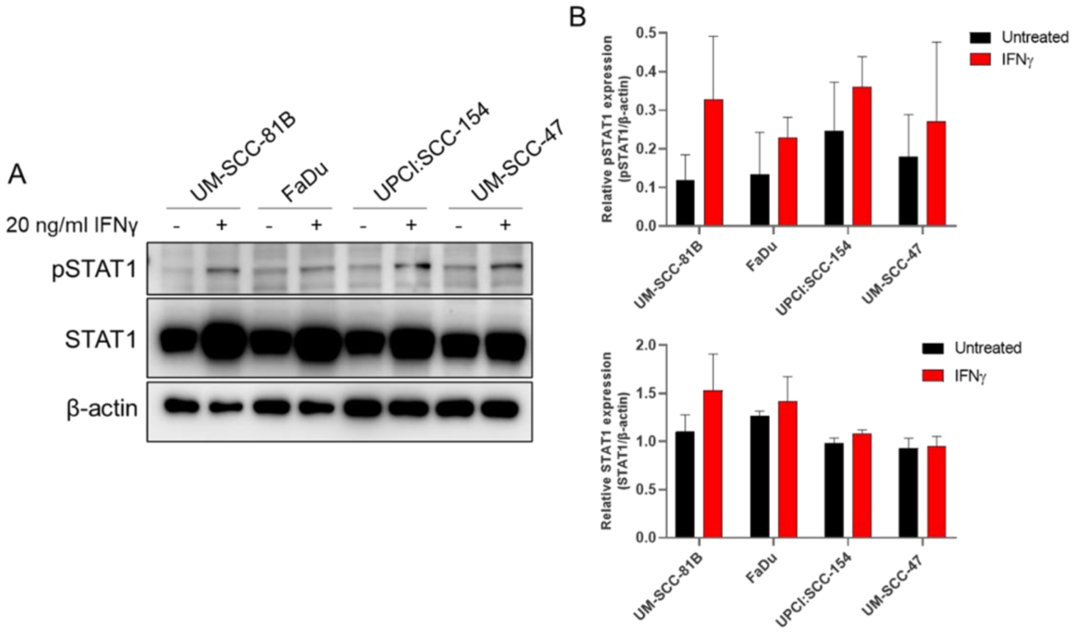

Supplementary figure 4. Validation of RNA-seq analysis in HPV+ and HPV- HNSCC cell line. A UM-SCC-81B and FaDu (HPV-) and UPCI:SSC-154 and UM-SCC-47 (HPV+) cells were treated with 20 ng/mL IFNγ for 24 h before analysis of phosphorylated and total STAT1 protein expression using Western blotting. **B** Densitometry plots represent the relative levels of phosphorylated and total STAT1 expression normalised against β-actin expression. Mean ± SD (n=3).
