# Supplementary material for: Differential Regulation of the STING Pathway in Human Papillomavirus–Positive and -Negative Head and Neck Cancers
Source: Cancer Res Commun. 2024 Jan 16;4(1):118–33. doi: 10.1158/2767-9764.CRC-23-0299 (PMC10793589; doi:10.1158/2767-9764.CRC-23-0299)
Supplement: Supplementary Figure 5 — demonstrates that HNSCC cells specifically inhibit PBMC-mediated IFNβ production. [file crc-23-0299-s05.pdf]

Supplemental Figure 5

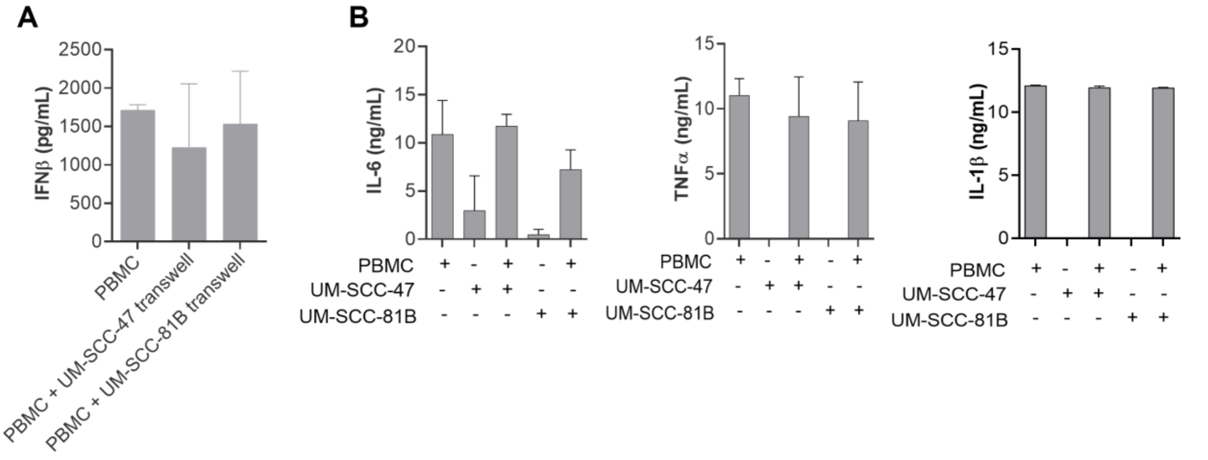

Supplementary figure 5. HNSCC cells specifically inhibit PBMC-mediated IFNβ production. A PBMCs (3x10^{6}) were stimulated for 6 h with 10 μg/mL CDA and co-cultured in the presence of HNSCC cells (2x10^{5}) in a transwell plate and culture supernatant examined for levels of INFβ by ELISA. **B** PBMCs (3x10^{6}) were stimulated for 6 h with 10 μg/mL CDA and co-cultured with UM-SCC-47 (HPV^{+}) or UM-SCC-81B (HPV^{-}) (2x10^{5}) and TNFα, IL-1β and IL-6 in culture supernatant measured by ELISA (n=3 independent experiments).
