# Supplementary material for: Differential Regulation of the STING Pathway in Human Papillomavirus–Positive and -Negative Head and Neck Cancers
Source: Cancer Res Commun. 2024 Jan 16;4(1):118–33. doi: 10.1158/2767-9764.CRC-23-0299 (PMC10793589; doi:10.1158/2767-9764.CRC-23-0299)
Supplement: Supplementary Figure 6 — shows the viability of HNSCC cells following STING stimulation and PBMC co-culture. [file crc-23-0299-s06.pdf]

Supplemental Figure 6

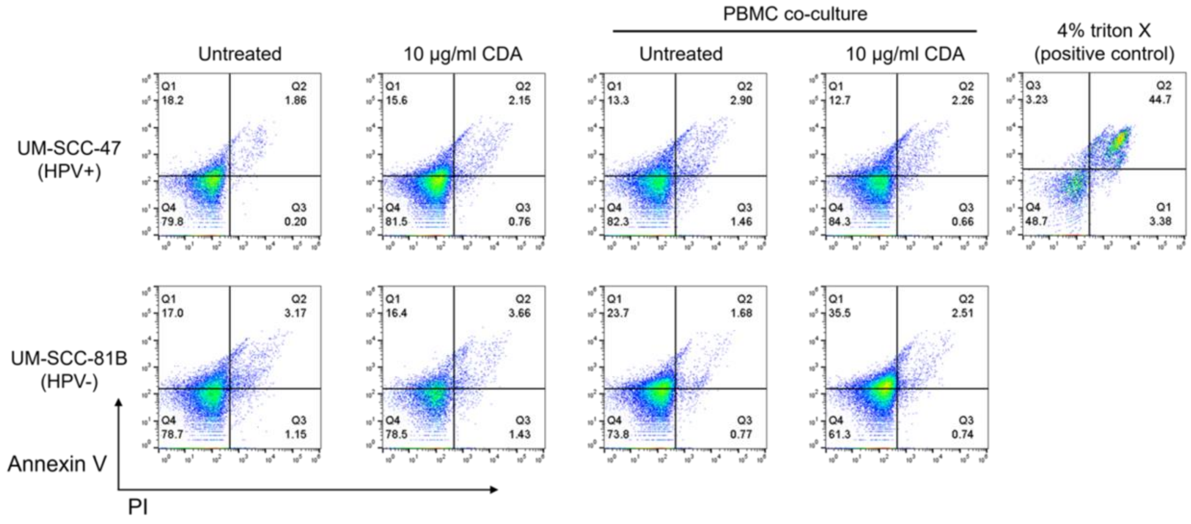

Supplementary figure 6. Viability of HNSCC cells following CDA treatment and PBMC co-culture. Representative scatter plots of UM-SCC-47 (HPV^+) and UM-SCC-81B (HPV^-) cells stained with Annexin V and PI following 6 h co-culture with PBMCs ± 10 µg/mL CDA stimulation (n=2). Annexin V positive cells represent apoptotic cells, double stain represents dead cells.
