# Supplementary material for: Differential Regulation of the STING Pathway in Human Papillomavirus–Positive and -Negative Head and Neck Cancers
Source: Cancer Res Commun. 2024 Jan 16;4(1):118–33. doi: 10.1158/2767-9764.CRC-23-0299 (PMC10793589; doi:10.1158/2767-9764.CRC-23-0299)
Supplement: Supplementary Figure 7 — shows how immune cell populations were identified from mass cytometry data. [file crc-23-0299-s07.pdf]

Supplemental Figure 7

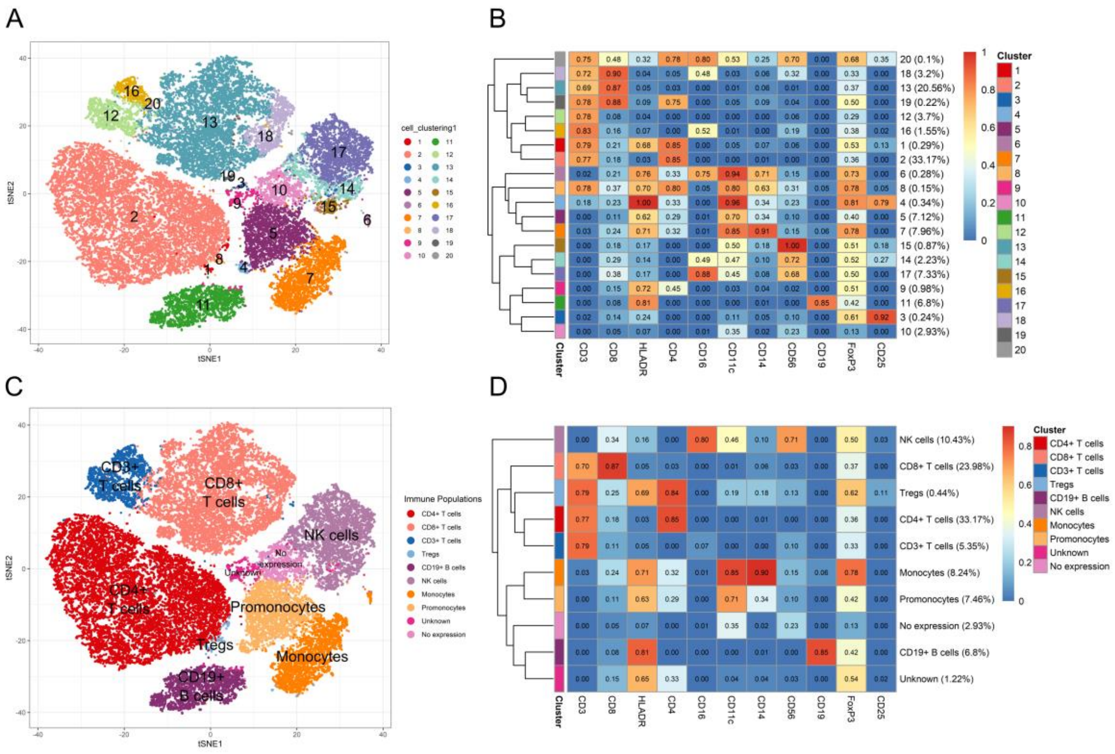

Supplementary figure 7. Identification and expression profiles of immune cell populations from mass cytometry data. *FlowSom* and *ConsensusClusterPlus* were used for unsupervised clustering of CD45^{+} cells into 20 subsets based on the relative expression of lineage markers. Visualisation of the clusters in a **A** t-SNE plot and **B** heatmap aided with manual merging of the metaclusters into defined immune cell populations. User-defined immune cell populations were visualised and validated in a **C** t-SNE plot and **D** heatmap.
