# Supplementary material for: Differential Regulation of the STING Pathway in Human Papillomavirus–Positive and -Negative Head and Neck Cancers
Source: Cancer Res Commun. 2024 Jan 16;4(1):118–33. doi: 10.1158/2767-9764.CRC-23-0299 (PMC10793589; doi:10.1158/2767-9764.CRC-23-0299)
Supplement: Supplementary Figure 8 — shows the viability of PBMCs following STING stimulation and HNSCC co-culture. [file crc-23-0299-s08.pdf]

Supplemental Figure 8

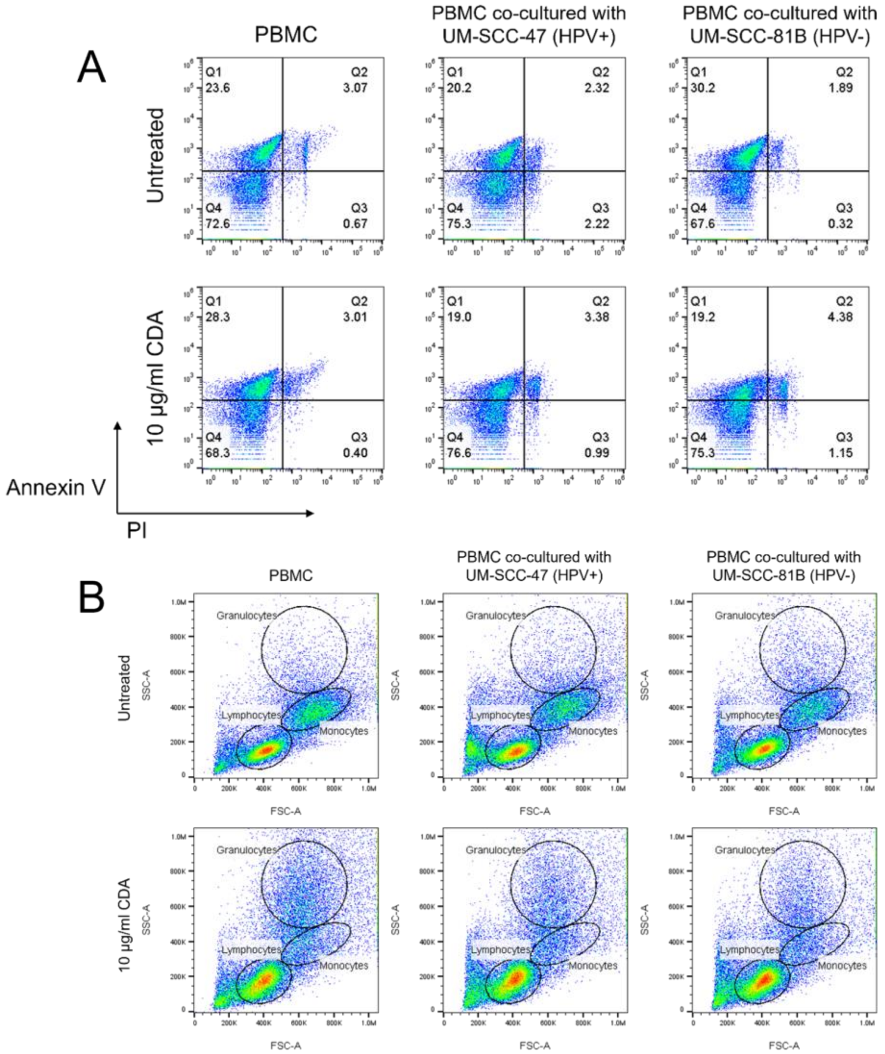

Supplementary figure 8. Viability of PBMCs following CDA stimulation. A. Representative scatter plots of healthy donor PBMCs following 6 h co-culture with UM-SCC-47 (HPV^{+}) or UM-SCC-81B (HPV^{-}) cells ± 10 µg/mL CDA stimulation stained with annexin V and propidium iodide (PI). Annexin V positive cells represent apoptotic cells, double stain represents dead cells. **B**. Representative scatter plots of healthy donor PBMCs either alone or following 6 h co-culture with UM-SCC-47 or UM-SCC-81B cells ± 10 µg/mL CDA. Gates are shown for lymphocyte, monocyte, and granulocyte populations.
