# Supplementary material for: Differential Regulation of the STING Pathway in Human Papillomavirus–Positive and -Negative Head and Neck Cancers
Source: Cancer Res Commun. 2024 Jan 16;4(1):118–33. doi: 10.1158/2767-9764.CRC-23-0299 (PMC10793589; doi:10.1158/2767-9764.CRC-23-0299)
Supplement: Supplementary Figure 9 — shows the surface level expression of EGFR across the HNSCC cell lines. [file crc-23-0299-s09.pdf]

Supplemental Figure 9

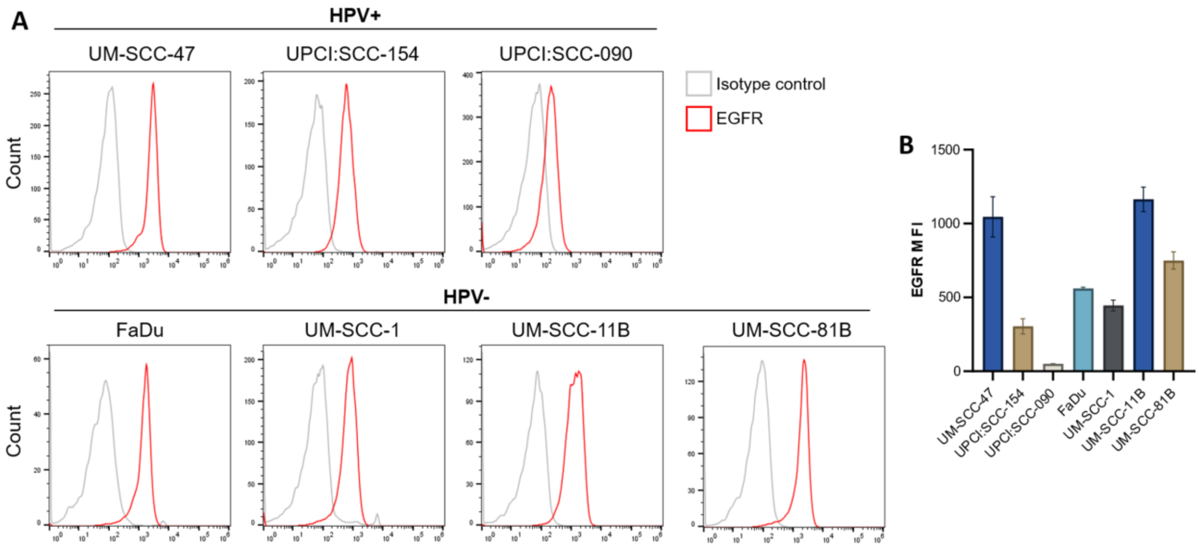

Supplementary figure 9. Characterisation of EGFR surface expression in HNSCC cell lines. A Representative flow cytometry histograms of basal EGFR expression in various HPV^{+} and HPV^{-} HNSCC cell lines. **B** Bar chart summarises the average mean fluorescence intensity (MFI) ± SD for EGFR (n=4).
