# Supplementary material for: Differential Regulation of the STING Pathway in Human Papillomavirus–Positive and -Negative Head and Neck Cancers
Source: Cancer Res Commun. 2024 Jan 16;4(1):118–33. doi: 10.1158/2767-9764.CRC-23-0299 (PMC10793589; doi:10.1158/2767-9764.CRC-23-0299)
Supplement: Supplementary Figure 10 — provides validation of the STING antibody used for IHC. [file crc-23-0299-s10.pdf]

Supplemental Figure 10

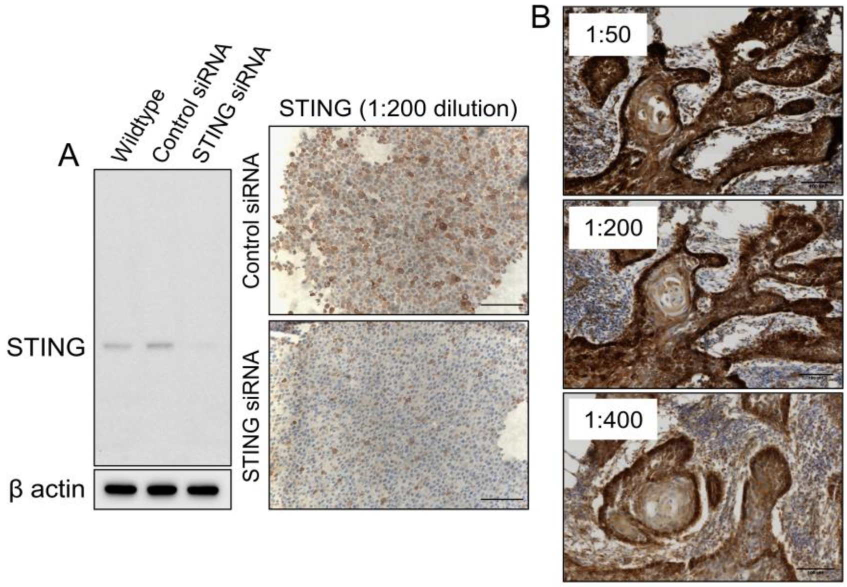

Supplementary figure 10. Validation of the STING antibody for IHC analysis. A Immunoblotting and IHC analysis of paraffin-embedded sections of FaDu cells following transfection with control- or STING-targeting siRNA (15 µM) were used to assess the specificity of the STING antibody. **B** HNSCC patient tissue sections were stained with various dilutions (1:50, 1:200 and 1:400) of the STING antibody to determine the optimum dilution for TMA staining (1:400 used for TMA staining).
