# Supplementary material for: Differential Regulation of the STING Pathway in Human Papillomavirus–Positive and -Negative Head and Neck Cancers
Source: Cancer Res Commun. 2024 Jan 16;4(1):118–33. doi: 10.1158/2767-9764.CRC-23-0299 (PMC10793589; doi:10.1158/2767-9764.CRC-23-0299)
Supplement: Supplementary Figure 11 — shows the whole slide images of the TMA sections chosen as representative images. [file crc-23-0299-s11.pdf]

Supplemental Figure 11

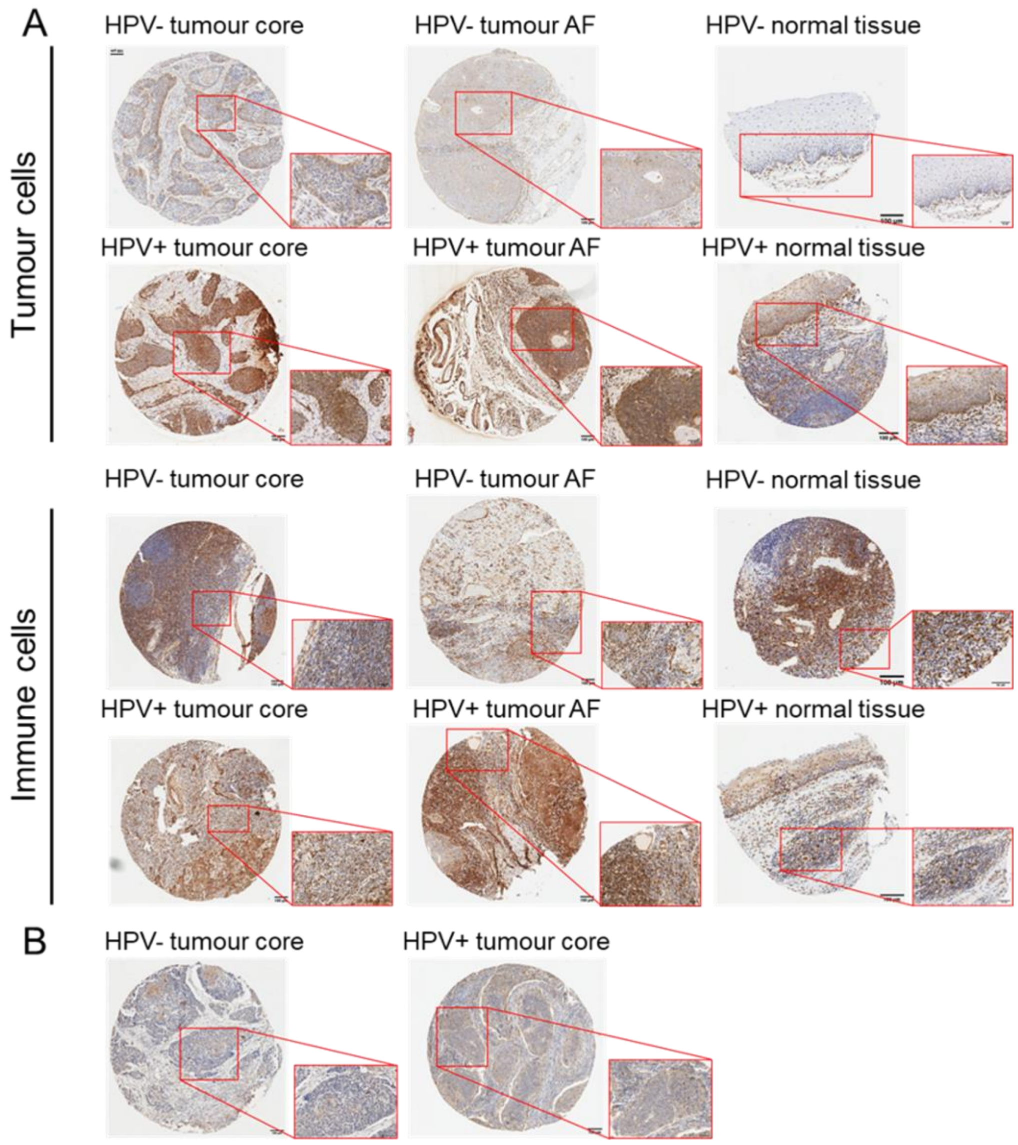


**Supplementary figure 11. Whole slide images of TMA sections.** Whole slide images of tissue core sections used as representative images for **A** STING and **B** IFNβ staining of the oropharyngeal TMAs. Scale bars = 100 μm for whole slide image and 50 μm for representative images.
