# Supplementary material for: Differential Regulation of the STING Pathway in Human Papillomavirus–Positive and -Negative Head and Neck Cancers
Source: Cancer Res Commun. 2024 Jan 16;4(1):118–33. doi: 10.1158/2767-9764.CRC-23-0299 (PMC10793589; doi:10.1158/2767-9764.CRC-23-0299)
Supplement: Supplementary Table 1 — shows the antibodies and recombinant proteins used in the study. [file crc-23-0299-s12.docx]

**Supplementary table 1. Antibodies and recombinant proteins used in the study**

| **Antibody/Protein** | **Clone** | **Supplier (Cat. No)** | **Usage** |
| --- | --- | --- | --- |
| Anti-phospho-STING (Ser366) | D7C3S | Cell Signaling (19781) | WB |
| Anti-STING | D2P2F | Cell Signaling (13647) | WB/IHC |
| Anti-phospho-TBK1 (Ser172) | D52C2 | Cell Signaling (5483) | WB |
| Anti-TBK1 | E8I3G | Cell Signaling (38066) | WB |
| Anti-phospho-IRF3 (Ser396) | D6O1M | Cell Signaling (29047) | WB |
| Anti-IRF3 | D83B9 | Cell Signaling (4302) | WB |
| Anti-MAVS | D5A9E | Cell Signaling (24930) | WB |
| Anti-MyD88 | D80F5 | Cell Signaling (4283) | WB |
| Anti-β-actin | AC-15 | Sigma-Aldrich (A1978) | WB |
| Anti-STAT1 |  | Proteintech (10144-2-AP) | WB |
| Anti-phospho-STAT1 (Ser727) |  | Proteintech (28977-1-AP) | WB |
| Anti-rabbit IgG, HRP-linked |  | Cell Signaling (7074) | WB |
| Anti-mouse IgG, HRP-linked |  | Cell Signaling (7076) | WB |
| Anti-IFNβ |  | Invitrogen (PA5-20390) | IHC |
| pcDNA3.1 STING plasmid | OHu16678D | GenScript (NM_198282.4) | STING overexpression |
| Hs_TMEM173_2 siRNA |  | Qiagen (SI04263189) | siRNA |
| Negative Control siRNA |  | Qiagen (1027280) | siRNA |
| Anti-human-CD3 | OKT3 | Biolegend (317318) | Functional studies |
| Anti-human-CD28 | CD28.2 | Biolegend (302902) | Functional studies |
| Anti-human-CD8-APC | SK1 | Biolegend (344722) | Flow cytometry |
| Anti-human-CD107a-PE | H4A3 | Biolegend (328608) | Flow cytometry |
| Anti-human-CD56-APC | CMSSB | Thermo Fisher (17-0567-42) | Flow cytometry |
| Anti-human-CD3-FITC | OKT3 | Biolegend (300306) | Flow cytometry |
