# Supplementary material for: Differential Regulation of the STING Pathway in Human Papillomavirus–Positive and -Negative Head and Neck Cancers
Source: Cancer Res Commun. 2024 Jan 16;4(1):118–33. doi: 10.1158/2767-9764.CRC-23-0299 (PMC10793589; doi:10.1158/2767-9764.CRC-23-0299)
Supplement: Supplementary Table 2 — details the antibodies used for mass cytometry. [file crc-23-0299-s13.docx]

**Supplementary table 2. Mass cytometry antibodies**

| **Antigen** | **Conjugate** | **Clone** | **Company (Cat. No)** | **Dilution** |
| --- | --- | --- | --- | --- |
| CD45 | 89Y | HI30 | Biolegend (304045) | 1/100 |
| CD45 | 106Cd | HI30 | Biolegend (304045) | 1/100 |
| CD45 | 110Cd | HI30 | Biolegend (304045) | 1/100 |
| CD45 | 115Cd | HI30 | Biolegend (500339) | 1/100 |
| IL-2 | 141Pr | MQ1-17H12 | Biolegend (304045) | 1/100 |
| CD19 | 142Nd | HIB19 | Standard Biotools (3142001B) | 1/200 |
| Granzyme B | 144Nd | GB11 | Standard Biotools (3171002B) | 1/400 |
| CD4 | 145Nd | RPA-T4 | Standard Biotools (3145001B) | 1/200 |
| CD8 | 146Nd | RPA-T8 | Standard Biotools (3146001B) | 1/200 |
| CD11c | 147Sm | Bu15 | Standard Biotools (3147008B) | 1/100 |
| CD14 | 148Nd | RMO52 | Standard Biotools (3148010B) | 1/100 |
| CD56 | 149Sm | NCAM16.2 | Standard Biotools (3149021B) | 1/100 |
| TNFα | 153Eu | Mab11 | Biolegend (502941) | 1/100 |
| PD-1 | 155Gd | EH12.2H7 | Biolegend (329941) | 1/100 |
| IL-10 | 159Tb | JES3-9D7 | Standard Biotools (3166008B) | 1/100 |
| CTLA-4 | 161Dy | 14D3 | Standard Biotools (3161004B) | 1/100 |
| CD69 | 162Dy | FN50 | Biolegend (310939) | 1/100 |
| FoxP3 | 164Dy | PCH101 | Life Technologies (14-4776-82) | 1/100 |
| Ki67 | 168Er | Ki-67 | Standard Biotools (350523) | 1/100 |
| CD25 | 169Tm | 2A3 | Standard Biotools (3169003B) | 1/100 |
| CD3 | 170Er | UCHT1 | Standard Biotools (3170001B) | 1/100 |
| Perforin | 173Yb | B-D48 | Standard Biotools (3175004B) | 1/200 |
| HLA-DR | 174Yb | L243 | Standard Biotools (3174001B) | 1/100 |
| CD16 | 209Bi | 3G8 | Standard Biotools (3209002B) | 1/100 |
